# The start of migration correlates with arrival timing, and the total speed of migration increases with migration distance in migratory songbirds: a cross-continental analysis

**DOI:** 10.1186/s40462-019-0169-1

**Published:** 2019-08-12

**Authors:** Heiko Schmaljohann

**Affiliations:** 1 Faculty of Biology/Environmental Sciences, University Oldenburg, 26111 Oldenburg, Germany; 2 Institute of Avian Research “Vogelwarte Helgoland”, An der Vogelwarte 21, 26386 Wilhelmshaven, Germany

**Keywords:** Anthropogenic changes, Arrival timing, Migration, Causes, Songbird, Start of migration, Total migration distance, Total speed of migration

## Abstract

**Background:**

Anthropogenic changes in the climate and environment have globally affected ecological processes such that the spatiotemporal occurrence of the main annual cycle events (i.e., breeding, wintering, moulting, and migration) has shifted in migratory birds. Variation in arrival timing at migratory destinations can be proximately caused by an altered start of migration, total migration distance, and/or total speed of migration. Quantifying the relative contributions of these causes is important because this will indicate the mechanisms whereby birds could potentially adjust their annual cycle in response to global change. However, we have relatively little quantitative information about how each of these factors contributes to variation in arrival timing. My main aims are to estimate how arrival timing is correlated with variation in the start of migration and the total migration distance and how the total speed of migration may change with the total migration distance and body mass in a comprehensive analysis including multiple species.

**Methods:**

For this purpose, I considered individual tracks covering complete migrations from multiple species and distinguished between within- and between-species effects.

**Results:**

Assuming that the within- and between-species effects quantified under this approach agree with the effects acting at the individual level, starting migration one day later or increasing the total migration distance by 1000 km would result in later arrival timing by 0.4–0.8 days or 2–5 days, respectively. The generality with which the start of migration is correlated with arrival timing within species suggests that this is the general biological mechanism regulating arrival timing, rather than the total migration distance. The total speed of migration was positively correlated with the total migration distance but not with the bird’s body mass.

**Conclusions:**

As the start of migration is endogenously controlled and/or affected by hatching date, directional selection can probably act on existing within-species/within-population variation to alter arrival timing. This factor and the importance of variation in the start of migration for arrival timing suggest that migratory species/populations in which there is sufficient variation in the start of migration and transgenerational processes affect the corresponding timing may present an advantage over others in coping with anthropogenic-induced global changes.

**Electronic supplementary material:**

The online version of this article (10.1186/s40462-019-0169-1) contains supplementary material, which is available to authorized users.

## Background

Migrant birds cope with seasonal variation in the environment by breeding and wintering in habitats that are temporarily favourable for their specific requirements. In addition to breeding, moulting, and wintering, seasonal movements account for a significant part of migrants’ annual cycle in terms of energy expenditure and time [1]. Birds’ breeding strategies, moulting schemes, and migrations are therefore well matched and adapted to the ecosystems they inhabit within their annual cycle [2]. Anthropogenic-induced changes in climate and environment have altered ecosystems, significantly affecting ecological processes such that the temporal and spatial occurrence of essential resources has globally shifted [3]. These spatiotemporal changes have modified the seasonal timing of migrants’ annual cycles [4–7], which may reduce reproductive success (e.g., when migrants arrive late and miss the time of optimal food abundance for chick rearing) [8, 9].

Variation in arrival timing can occur because of variation in three migratory traits (among others): the start of migration, the total migration distance, and the total speed of migration (i.e., the total migration distance [km] covered per unit of time [day], including periods of stopover) [10]. For different species and seasons, it has been demonstrated in several studies that (i) the earlier an individual starts its migration, the earlier it is expected to arrive at its migratory destination [11–16]; (ii) the longer the total migration distance, the later the bird is expected to arrive at its migratory destination, given that the two other traits remain unchanged [17]; and (iii) birds with a higher total speed of migration arrive earlier than those with a lower speed [12]. Furthermore, the total speed of migration is positively correlated with the total migration distance [18, 19] and negatively correlated with body mass [20]. The latter correlation has been shown for parts of the seasonal migratory movements of different populations [21], for the total migration of seven sandpiper species in spring [22], and in general for flapping and soaring migrants [23]. Quantifying the relative potential contributions of variations in these traits to variation in arrival timing and to what degree the total migration distance and body mass affect the total speed of migration in a multiple species approach to identify within- and between-species effects is still a major challenge in ecology.

Here, I focus on migrant songbirds, a group that has been widely studied in relation to anthropogenic changes [24–26]. The first objective is to jointly quantify the relative contributions of the start of migration and total migration distance to variation in arrival timing separately for spring and autumn. For statistical reasons, the total speed of migration was not included because it is an arithmetic function of the start of migration, arrival timing, and total migration distance and would therefore lead to spurious results. I consider individual migration data detailing movements for complete migratory seasons and distinguish between within-species and between-species effects [27]. By quantifying the relative contributions of the start of migration and total migration distance to the variation in arrival timing, we can carefully predict how much a certain change in one of these migratory traits potentially affects arrival timing given that the other remains unchanged. This is a simplification because the start of migration may affect the total speed of migration [28] and current environmental conditions influence the start of migration [29] and the total speed of migration [30, 31], probably evoking more complex reactions that are limited by species-specific constraints on migration [32]. However, it allows us to cautiously discuss the magnitude of adjustment that would be required to separately explain the 0.2- to 1-day per year advance in breeding area arrival timing observed in many migrant songbirds (i.e., 4 to 20 days in the last 20 years) [5, 6, 33, 34].

The second objective is to quantify the observed effect of the total migration distance [18, 19] and the predicted effect of body mass [20] on the total speed of migration. Since the total speed of migration is an arithmetic function of the total migration distance, one may expect the latter to be positively correlated with the total speed of migration, cf. La Sorte et al. [21]. Body mass has been negatively correlated with the total speed of migration in three studies [21–23]. However, it remains unknown whether these patterns may influence the individual total speed of migration for songbirds in a similar way when accounting for complete migration periods.

**Fig. 1:**
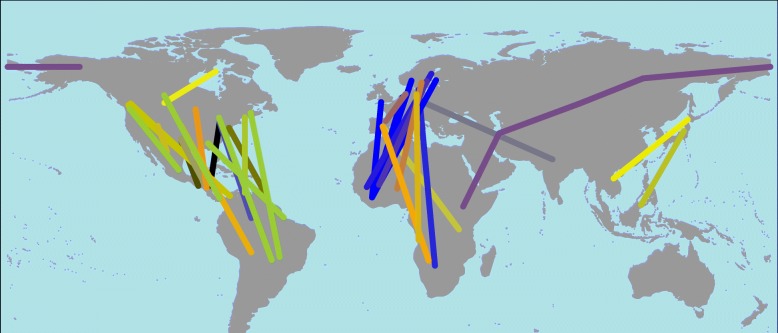
Simplified migration routes of the study species. Breeding areas and wintering grounds are connected by a straight line; thus, the “true” migration routes differ from the presented ones. For each species/population, the average longitude and latitude of the breeding area and wintering ground were considered. For pied flycatchers (*Ficedula hypoleuca*, orange), northern wheatears (*Oenanthe oenanthe*, green), and barn swallows (*Hirundo rustica*, grey), the location estimates are given for the population-specific breeding areas. The species-specific colours are given in Table 1

Despite recent advances in miniaturized tracking devices, the sample sizes of repeatedly tracked individual songbirds are still rather small [11, 35, 36]. At present, it is therefore not possible to determine within-individual effects on variation in arrival timing. As an alternative option, I assumed that the effects acting at the individual level are captured by between-individual comparisons within and across different species, cf. La Sorte et al. [21]. I considered published data for 26 different songbird species detailing the timing of the main annual cycle events and providing individual estimates of the total migration distance and total speed of migration across five continents (Table 1, Fig. 1). In assessing the above expectations with the individual tracking data, the main aims were to estimate within- and between-species effects of how arrival timing is correlated with variation in the start of migration and total migration distance and how the total speed of migration may change with the total migration distance and body mass in a multi-species approach.

**Table 1:** Details of the species and references used in this study. The common name, scientific name, appropriate colour, order, family, and sample sizes of the included birds (spring: spr; autumn: aut) and mean values for the start of migration (day of year; 1 January = 1), end of migration (day of year), total migration distance, and total speed of migration are given. Whether birds stayed predominately at one wintering ground (yes) or at more than one (no) and the lowest body mass indicated by the Handbook of the Birds of the World are detailed. The studies’ corresponding references are listed. Species were ordered based on the phylogenetic tree of the included species derived from TIMETREE (www.timetree.org, see above and Additional file 1). Studies on the Eurasian cuckoo (*Cuculus canorus*; family: Cuculidae) and the European Roller (*Coracias garrulus*; family: Coraciidae) were included in this study on Passeriformes (songbirds), as the migration ecology of these species is similar to that of songbirds. All raw data are provided in Additional file 3

No.	Common name	Scientific name	Colour	Order	Family	Sample size		Start of migration[day of year]		End of migration [day of year]		Total migration distance [km]		Total speed of migration [km/day]		One main wintering ground	Body mass [g]	Reference
						spr	aut	spr	aut	spr	aut	spr	aut	spr	aut			
1	barn swallow	*Hirundo rustica*		Passeri-formes	Hirundinidae	16	27	93	239	129	308	7933	7933	262	136	yes	16	[37]
2	chestnut-cheeked starling	*Sturnus philippensis*		Passeri-formes	Sturnidae	4	16	84	254	111	286	4117	4109	156	140	yes	50	[38]
3	northern wheatear (Alaska)	*Oenanthe oenanthe*		Passeri-formes	Muscicapidae	–	8	–	224	–	317	–	14,789	–	160	yes	18	[39]
4	northern wheatear (Sweden)	*Oenanthe oenanthe*		Passeri-formes	Muscicapidae	12	12	76	228	114	289	5247	5162	167	86	yes	18	[40]
5	Stejneger’s stonechat	*Saxicola stejnegeri*		Passeri-formes	Muscicapidae	–	12	–	282	–	336	–	4950	–	92	yes	13	[41]
6	pied flycatcher	*Ficedula hypoleuca*		Passeri-formes	Muscicapidae	8	14	94	227	112	268	5243	5390	316	140	yes	9.7	[42]
7	semicollared flycatcher	*Ficedula semitorquata*		Passeri-formes	Muscicapidae	10	11	48	202	98	285	5410	5775	114	69	yes	8	[43]
8	red-spotted bluethroat	*Luscinia s. svecica*		Passeri-formes	Muscicapidae	2	3	98	243	128	293	5425	6085	186	152	yes	12	[44]
9	Swainson’s thrush	*Catharus ustulatus*		Passeri-formes	Turdidae	37	39	97	218	148	297	2147	5561	118	84	yes	25	[45, 46]
10	veery	*Catharus fuscescens*		Passeri-formes	Turdidae	5	3	104	239	135	318	4530	6912	100	89	no	25	[47]
11	wood thrush	*Hylocichla mustelina*		Passeri-formes	Turdidae	5	–	109	–	125	–	3860	–	242	–	yes	40	[48]
12	great reed warbler	*Acrocephalus arundinaceus*		Passeri-formes	Acrocepha-lidae	6	8	103	216	134	256	7083	6286	220	160	no	22	[49]
13	blackpoll warbler	*Setophaga striata*		Passeri-formes	Parulidae	3	5	120	286	142	305	4350	3820	197	349	yes	9.7	[50]
14	golden-winged warbler	*Vermivora chrysoptera*		Passeri-formes	Parulidae	6	6	104	245	138	295	3831	3831	117	82	yes	7.2	[51]
15	tawny pipit	*Anthus campestris*		Passeri-formes	Motacillidae	3	6	61	223	127	270	4530	4233	100	85	no	17	[52]
16	chestnut-collared longspur	*Calcarius ornatus*		Passeri-formes	Calcariidae	4	6	74	276	116	317	1396	1318	34	36	yes	17	[53]
17	snow bunting	*Plectrophenax nivalis*		Passeri-formes	Emberizidae	18	20	130	268	151	302	2147	2661	118	79	yes	18	[54]
18	ortolan bunting	*Emberiza hortulana*		Passeri-formes	Emberizidae	6	6	95	234	136	290	6061	6061	146	106	yes	17	[55]
19	linnet	*Carduelis cannabina*		Passeri-formes	Fringillidae	5	6	45	258	109	325	1496	1489	24	24	yes	15	[56]
20	red-backed shrike	*Lanius collurio*		Passeri-formes	Laniidae	6	9	84	226	147	324	11,862	9719	190	99	yes	22.5	[57]
21	red-eyed vireo	*Vireo olivaceus*		Passeri-formes	Vireonidae	10	1	83	247	131	294	4814	5264	104	112	yes	12	[58]
22	willow warbler	*Phylloscopus trochilus*		Passeri-formes	Phylloscopidae	–	15	–	215	–	284	–	6058	–	92	yes	6.3	[59]
23	Eastern kingbird	*Tyrannus tyrannus*		Passeri-formes	Tyrannidae	8	8	110	245	132	288	5249	6437	264	128	yes	40	[60]
24	scissor-tailed flycatcher	*Tyrannus forficatus*		Passeri-formes	Tyrannidae	2	5	100	296	108	307	2515	2599	419	284	yes	39	[60]
25	Western kingbird	*Tyrannus verticalis*		Passeri-formes	Tyrannidae	6	11	110	204	126	211	2570	1425	278	219	no	35	[60]
26	European roller	*Coracias garrulus*		Coracii-formes	Coraciidae	4	10	64	228	122	308	9318	9259	168	97	yes	127	[61]
27	common cuckoo	*Cuculus canorus*		Cuculi-formes	Cuculidae	3	6	38	192	140	307	9136	7118	94	65	no	115	[62]

## Methods

### Field studies and study species

For the analyses, I considered studies individually tracking the start of the spring and/or autumn migration (departure from the wintering ground or breeding area, respectively), the corresponding arrival time at the migratory destination, the total migration distance, and the total speed of migration during at least one complete seasonal migration (Table 1). Only species with data for more than three individuals within one season were considered. Two studies involving species not belonging to the order of songbirds (Passeriformes), one in the Eurasian cuckoo (*Cuculus canorus*, Cuculiformes) and another in the European roller (*Coracias garrulus*, Coraciiformes), were included because the migration ecology of these species is similar to that of songbirds [61, 62]. Two populations of the northern wheatear (*Oenanthe oenanthe*) were tracked [39, 40]. I treated these populations separately (i.e., as different “species”) because of the apparent differences in their movement ecology and migration distance (from Sweden to western Africa [40] or from Alaska to eastern Africa [39]) (Table 1). The tawny pipit (*Anthus campestris*), the linnet (*Linaria cannabina*), and the snow bunting (*Plectrophenax nivalis*) are typical diurnal migrants, while the ortolan bunting (*Emberiza hortulana*) migrates during the night and during the day [63]. All the others are nocturnal migrants, but some may prolong their migratory flights into the day [64] and/or cross the Sahara Desert non-stop [64–67].

### Light-level geolocation

All birds were tracked by light-level geolocation. For any given site, light intensity changes specifically over the year with respect to a standard time, which allows positions to be estimated twice per day [68, 69]. The light-level data were analysed by different analytic procedures in the original studies, resulting in different yet unquantifiable levels of accuracy and precision for the location estimates. Light-level geolocators accordingly do not track daily fine-scale movements and provide only “inexact” approximations of the general migratory route [70, 71]. Furthermore, location estimates may be even less accurate at high altitudes during summer [71], especially if they are not analysed in a sophisticated way [72, 73]. To assess the accuracy and precision of the location estimates, many studies apply ground truthing in breeding areas or wintering grounds. However, these values cannot necessarily be generalized for other periods of the annual cycle in the considered studies, but see Rakhimberdiev et al. [74]. Habitat-specific shading characteristics and season-specific behaviour (e.g., barn swallows (*Hirundo rustica*) often roost in reed beds during the non-breeding period) both alter the actual recorded light intensity in comparison with the ground-truthing period [71]. Therefore, even if the accuracy and precision of the location estimates were provided in the original studies, they would not capture the uncertainties of the location estimates obtained away from the ground-truthing site.

In most of the included species, individuals over-wintered in one defined area (Table 1), while some individuals of the veery (*Catharus fuscescens*), the great reed warbler (*Acrocephalus arundinaceus*), the tawny pipit (*Anthus campestris*), the Western kingbird (*Tyrannus verticalis*), and the common cuckoo visited multiple areas during this period [47, 49, 52, 60, 62]. If the traits of interest were not individually detailed in the original publications, I considered the departure from the last wintering ground as the start of migration and the distance from the last wintering ground to the breeding area as the total migration distance for those individuals. Likewise, arrival at and the distance to the “first” wintering ground were considered to estimate the corresponding traits in autumn. In doing so, we obviously missed the first migratory fuelling period, which includes the energy accumulation and adjustment of organs (e.g., muscle) for migration [75] that takes place in the immediate vicinity of the breeding areas/wintering grounds. The total duration of migration is underestimated depending on the duration of this first migratory fuelling period, and consequently, the total speed of migration is overestimated. Migratory tracks derived from light data represent simplified and smoothed routes of real movements [70, 71]. Therefore, the total migration distance is always underestimated, and consequently, the total speed of migration is also underestimated. In the studies that did not provide the total migration distance, I simplified this parameter as the cumulative great circle distance between the breeding area, stopover sites, and wintering ground. The coordinates of the individual location estimates for individual birds were extracted from the studies or, when not given, were approximated from map locations using Google Earth, cf. Finch et al. [76]. In summary, the estimates of the total speed of migration used in this study are subject to some inaccuracies because both the total duration of migration and the total migration distance are underestimated. Because it is unlikely that these two inaccuracies will cancel each other out, we should keep in mind that the total speed of migration may be higher or lower when discussing the results.

The latitude of the wintering grounds was significantly correlated with the total migration distance (lm: 95% CrI: − 0.005 – − 0.003 °/km, *n* = 270), but the within-species variation in latitude was low and therefore was not considered.

### Body mass

The body mass of a migratory songbird can vary by up to or even more than 100% compared with lean conditions (i.e., without any migratory energy stores) over the course of the year [77]. Hence, the body mass measured at a certain date within a year in the considered studies does not provide a representative scale for the species’ speed of migration. I dealt with this issue by using the lowest body mass given in the corresponding species description in the Handbook of the Birds of the World (HBW) (e.g., del Hoyo, Elliott & Christie [78]) (Table 1). This approach using the species’ “lean body mass” eliminated the bias that occurs when body mass measurements are taken within the annual cycle and excluded potential species-specific differences in the physiological capacity to accumulate energy. However, the lowest HBW body mass estimate per species may not necessarily perfectly capture the true lean body mass of that species.

### Statistical analyses

The statistical analyses were implemented using R [79]. All data and R scripts needed to completely reproduce the analyses are made available (Additional files 1, 2 and 3).

The data include different species with measurements for different individuals within a species; thus, the data exhibit two levels of aggregation (i.e., within and between species). The measurements within a species are not independent because of species−/population-specific innate migration programmes [80, 81] and habitat/food requirements. To avoid the problem of pseudoreplication and to distinguish between within-species and between-species effects (i.e., to not erroneously generalize within-species effects to between-species effects or vice versa), I followed the statistical approach detailed by van de Pol & Wright [27].

The variation in arrival timing (*y*) among individuals of different species was modelled separately for spring and autumn using a linear mixed-effect model run with functions provided in the R package “*lme4*” [82] and assuming normally distributed errors. Arrival timing [Julian date] (*y*), the start of migration [Julian date] (*x*), and the total migration distance [km] (*z*) were each scaled separately for spring and autumn across all species. Within-species variation in the start of migration (*x*) and total migration distance (*z*) was captured by within-species centring performed separately for spring and autumn. Centring around species means effectively eliminates any between-species variation, which provides two new fixed effects expressing only the within-subject variation in the start of migration $\left({x}_{ij}-{\overline{x}}_j\right)$ and total migration distance $\left({z}_{ij}-{\overline{z}}_j\right)$. To express only the between-species variation, I generated two other new fixed predictor variables, which were simply the species’ means for the start of migration $\left({\overline{x}}_j\right)$ and the total migration distance $\left({\overline{z}}_j\right)$. Species were included in each model as a random intercept (*u*_0*j*_). To quantify the amount of between-species variation in within-species slopes around the start of migration, I added corresponding random slopes for the start of migration (*u*_*Wj*_). The inclusion of these random slopes allows the investigation of whether there is between-species variation in the slopes of the within-species effects of the start of migration [27]. I did not add random slopes for the total migration distance because between-individual variation within a species was low, and thus, including these random slopes would yield an overly complex random structure that would not be supported by the data. I applied the following regression equation to model the variation in arrival timing separately for spring and autumn:

1 $$ {y}_{ij}=\left({\beta}_0+{u}_{0j}\right)+\left({\alpha}_W+{u}_{Wj}\right)\left({x}_{ij}-{\overline{x}}_j\right)+{\alpha}_B{\overline{x}}_j+\left({\gamma}_W\right)\left({z}_{ij}-{\overline{z}}_j\right)+{y}_B{\overline{z}}_j+{e}_{0 ij} $$

with the intercept, *β*_0_, the within-species effect of the start of migration, *α*_*W*_, the between-species effect of the start of migration, *α*_*B*_, the within-species effect of the total migration distance, *γ*_*W*_, and the between-species effect of total migration distance, *y*_*B*_. The random intercept, *u*_0*j*_, the random slope, *u*_*Wj*_, and the residual term, *e*_0*ij*_, are assumed to be drawn from a normal distribution with a mean of zero and between-species variance of ${\upsigma}_{u_{Oj}}^2,$ between-species variance of ${\upsigma}_{u_{Wj}}^2,$ and within-species variance ${\upsigma}_{e_{0 ij}}^2,\mathrm{respectively}.$ The explanatory variables of each model were all tested against one another within each species for collinearity with the “vif” function in the R package “*usdm*” [83]. If the collinearity (variance inflation factor) was lower than 3, then the explanatory variables were treated as not collinear [84]. The explanatory variables were collinear in six species in spring and in four species in autumn. These species were excluded from the two corresponding models (Additional file 2).

The variation in the total speed of migration [km/d] (*y*) among individuals of different species was modelled using a linear mixed-effect model and assuming normally distributed errors. Here, the species that were excluded due to collinear explanatory variables in the above two models were included (Additional file 2). Since not all individuals and species were tracked in both seasons, I ran the model separately for spring and autumn. The total speed of migration (*y*) and total migration distance (*z*) were both log10-transformed. By following the statistical approach described above, one new fixed effect expressing only the within-subject variation in the total migration distance $\left({z}_{ij}-{\overline{z}}_j\right)$ and one expressing only the between-species variation in the total migration distance $\left({\overline{z}}_j\right)$ were generated. Species was included in each model as a random factor to allow random intercepts for the total migration distance (*v*_0*j*_). To quantify the amount of between-species variation in within-species slopes around the total migration distance, I added corresponding random slopes for the total migration distance (*v*_*Wj*_) [27]. I applied the following regression equation to model the variation in the total speed of migration separately for spring and autumn:

2 $$ {y}_{ij}=\left({\beta}_0+{v}_{0j}\right)+\left({\gamma}_W+{v}_{Wj}\right)\left({z}_{ij}-{\overline{z}}_j\right)+{y}_B{\overline{z}}_j+{e}_{0 ij} $$

with the intercept, *β*_0_, the within-species effect of the total migration distance, *γ*_*W*_, and the between-species effect of the total migration distance, *y*_*B*_. The random intercept, *v*_0*j*_, the random slope, *v*_*Wj*_, and the residual term, *e*_0*ij*_ are assumed to be drawn from a normal distribution with a mean of zero and between-species variance of ${\upsigma}_{v_{Oj}}^2,$ between-species variance of ${\upsigma}_{v_{Wj}}^2,$ and within-species variance of ${\upsigma}_{e_{0 ij}}^2,$ respectively.

To model the variation in the total speed of migration in relation to body mass, individual tracking data were aggregated based on the species/population, breeding latitude, and season to estimate the corresponding mean values. For the pied flycatcher and the barn swallow, different populations exhibited substantially different total migration distances, and these species were therefore split into different populations for this analysis. The variation in the total speed of migration between different species/populations was modelled using a linear mixed-effect model assuming normally distributed errors. The body mass of each species/population and the season and their corresponding two-way interaction were used as explanatory variables. All numeric variables were log10 transformed. The bird family was included as a random factor (intercept) to account for phylogenetic non-independence [21]. To account for differences in sample size and in the accuracy and precision of estimates between species/populations, the inverse of the standard error of the total speed of migration estimate was included as a species−/population-specific weight in the model. The two-way interaction was not significant and, thus, was omitted from the final model.

Comparative analyses including different species require control for the effect of the species’ phylogenetic relationships. As multivariate regressions considering sampling errors of more than one explanatory variable are, to the best of my knowledge, not yet available [85], I could not account for shared ancestry. Nevertheless, to model the variation in the total speed of migration, I ran a generalized least squares (GLS) regression model accounting for the phylogenetic relationships between species by including a within-group correlation structure defined by the phylogenetic tree of the species being considered (Additional file 1). The results were in agreement with the outcome of the model of the total speed of migration in relation to body mass (Additional file 2).

The diagnostic residual and random effect plots of all models indicated that the data did not violate the model assumptions (Additional file 2). To assess the uncertainty of the model estimates and model predictions, I used Bayesian methods to obtain uncertainty estimates of the model parameters. In all models, I used improper prior distributions: *p(β) ~ 1* for the coefficients and *p(β) ~ 1/σ* for the variance parameters, following Korner-Nievergelt et al. [86] and using the corresponding R package “*blmeco*”. To obtain the posterior distribution, I directly simulated 5000 values from the joint posterior distribution of the model parameters using the function *sim* of the R package “*arm*” [87]. The means of the simulated values from the joint posterior distributions of the model parameters were used as estimates, and the 2.5 and 97.5% quantiles were used as lower and upper limits of the 95% credible intervals (CrI). I declared an effect to be significant if the corresponding 95% CrI did not include zero or the 95% CrIs of the compared groups did not overlap. Within-species effects (*α*_*W*_, *γ*_*W*_) were treated as being significantly different from the corresponding between-species effects (*α*_*B*_, *γ*_*B*_) if the corresponding 95% CrIs did not overlap.

## Results

**Fig. 2:**
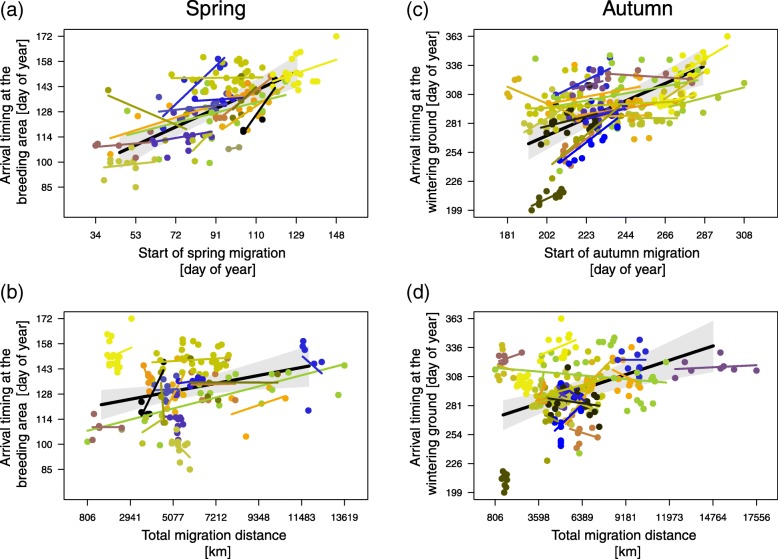
Model estimates of the between-species effects of the start of migration and the total migration distance on arrival timing at the migratory destination. **a** and **b** Arrival timing at the breeding area (161 individuals of 17 different species) and (**c** and **d**) at the wintering ground (241/21) as a function of the start of migration (**a** and **c**) and the total migration distance (b and d). To show the predicted between-species effect of each explanatory variable on arrival timing separately, the other variable was set to its corresponding mean value. The fitted values (black solid lines) with the 95% credible intervals (grey polygons) are given. The within-species effect of either explanatory variable on the dependent variable is visualized with species-specific regression lines, and the models’ corresponding random effects are shown in Fig. 3. Raw data are given in species-specific colours (Table 1)

**Fig. 3:**
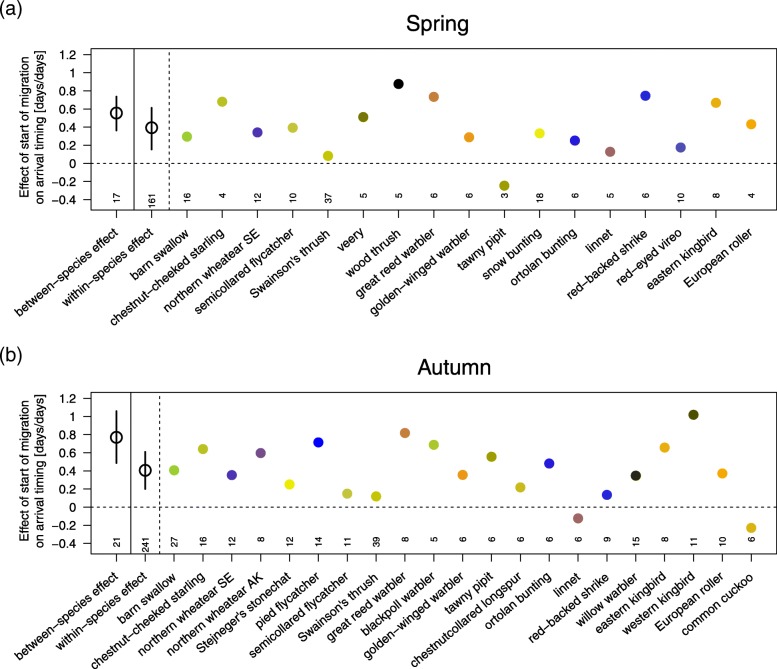
Between-species, within-species, and species-specific effects of the start of migration on arrival timing. For spring (**a**) and autumn (**b**), back-transformed between-species (with 95% credible intervals, CrI), within-species (with 95% CrI), and species-specific effects of the start of migration on arrival timing. Based on the random-factor structure, species-specific slopes for the explanatory variable were calculated by adding the species-specific random slope estimate and the bird family-specific random intercept estimate to the overall estimate of the corresponding explanatory variable (Additional file 2). Species were ordered based on the phylogenetic tree of the included species derived from TIMETREE (http://timetree.org, Additional file 1). Corresponding individual sample sizes are provided above the x-axis

The spring model of variation in arrival timing included 17 bird species (161 individuals), and the autumn model included 21 species (241 individuals). The start of migration was significantly and positively correlated with arrival timing within and between species in both seasons (Table 2; Fig. 2). The species differed in how strongly their start of migration was associated with their arrival timing, but for all but three species, the effect was positive and below 1 (Fig. 3). The back-transformed slopes demonstrated that varying the start of migration by 1 day would potentially shift arrival timing by approximately 0.4 days within species (both spring and autumn) and by 0.6 (breeding area) and 0.8 days (wintering ground) between species (Table 2).

**Table 2:** Results of the spring and autumn models of arrival timing. The within- and between-species effects of the start of migration and total migration distance on arrival timing at the breeding areas (n_species/populations_ = 17; n_individuals_ = 161) and wintering grounds (n_species/populations_ = 21; n_individuals_ = 241) in migrant songbirds. Numeric variables were scaled. Significant effects are displayed in bold. To evaluate the biological effects of the estimates, the corresponding slopes were back-transformed into the original units of the equivalent traits (*effect*)

Fixed effects	Modelling breeding area arrival timing			Modelling wintering ground arrival timing		
	Estimate	95% CrI	Effect [Julian date / unit of migratory trait]	Estimate	95% CrI	Effect [Julian date / unit of migratory trait]
*β*_0_ (intercept) [Julian date]	**0.51**	**0.36–0.65**	–	**0.29**	**0.04–0.54**	–
*α*_*W*_ (within-species effect of the start of migration) [Julian date]	**0.28**	**0.12–0.43**	0.39	**0.33**	**0.16–0.49**	0.41
*γ*_*W*_ (within-species effect of the total migration distance) [km]	**0.10**	**0.06–0.14**	0.0023	0.02	−0.01 – 0.06	0.0012
*α*_*B*_ (between-species effect of the start of migration) [Julian date]	**0.40**	**0.27–0.53**	0.56	**0.62**	**0.39–0.85**	0.77
*γ*_*B*_ (between-species effect of the total migration distance) [km]	**0.10**	**0.03–0.17**	0.0021	**0.10**	**0.05–0.16**	0.0048
Random effects						
Groups	Names	Std. dev.			Std. dev.	
Study species	intercept	0.064			0.065	
	Slope: within-species effect of the start of migration	0.244			0.263	
Residuals		0.055			0.048	
Marginal R^2^		0.54			0.39	
Conditional R^2^		0.82			0.79

The total migration distance was positively correlated with arrival timing within and between species in both seasons, though it was not significantly associated with wintering ground arrival timing within species (Table 2; Fig. 2). According to the back-transformed slopes, a 1000 km-shorter/longer migration distance would potentially shift arrival timing by approximately 2 days for the breeding area (within and between species) and by approximately 5 days for the wintering ground (only between species) (Table 2).

**Fig. 4:**
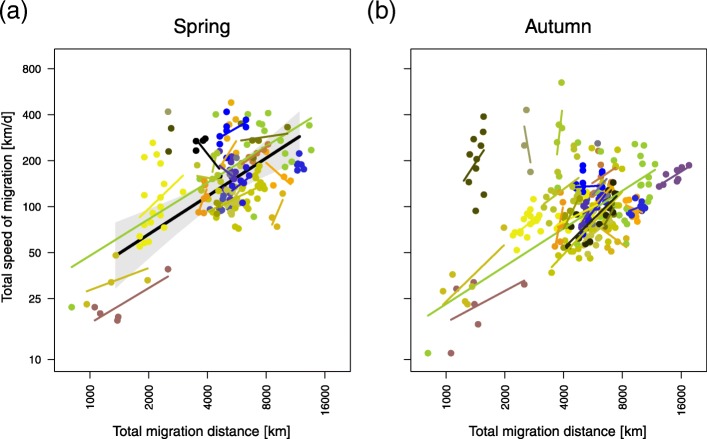
Model estimates of the between-species effect of the total migration distance on the total speed of migration. **a** Total speed of migration in spring (180 individuals of 22 species) and **b** in autumn (245/24) as a function of the total migration distance. The predicted between-species effect of the total migration distance on the total speed of migration is shown. The fitted values (black solid lines) with the 95% credible interval (grey polygon) are given. The within-species effects are visualized with species-specific regression lines. Raw data are given in species-specific colours (Table 1). The axes show the log10 transformed values of the corresponding variables

The total migration distance was significantly and positively correlated with the total speed of migration within species in both seasons, while the between-species effect was only significant in spring (Table 3; Fig. 4). Back-transforming the average between-species effect of the spring model clarified that an increase in the total migration distance of 100 km was associated with an increase in the total speed of migration by 3 km/d for “short” total migration distances (1300 km) and by 2 km/d for “long” total migration distances (10,000 km) (Fig. 4). In autumn, the within- and between-species effects differed significantly from each other, with a stronger effect being observed within species (Table 3).

**Table 3:** Results of the model of the total speed of migration. The within- and between-species effect of the total migration distance on the total speed of migration in migrant songbirds (spring: n_species/populations_ = 22; n_individuals_ = 180; autumn: n_species/populations_ = 24; n_individuals_ = 245). Numeric variables were log10 transformed. Significant effects are displayed in bold

Fixed effects	Modelling the total speed of migration in spring		Modelling the total speed of migration in autumn	
	estimate	95% CrI	estimate	95% CrI
Intercept	−0.92	−2.25 – 0.42	0.74	−0.59 – 2.07
*γ*_*W*_ (within-species effect of the total migration distance) [km]	**0.73**	**0.54–0.92**	**0.96**	**0.72–1.19**
*γ*_*B*_ (between-species effect of the total migration distance) [km]	**0.83**	**0.47–1.19**	0.34	−0.02 – 0.70
Random effects				
Intercept	0.191		0.214	
Slope: within-species effect of the total migration distance	0.047		0.156	
Residuals	0.133		0.135	
Marginal-R^2^	0.39		0.18	
Conditional-R^2^	0.80		0.77

**Fig. 5:**
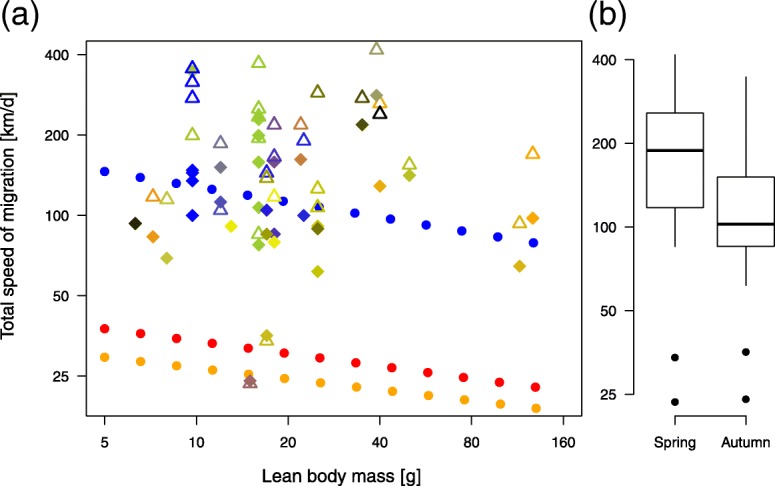
Total speed of migration in spring and autumn against “lean” body mass. **a** Population-specific average total speed of migration for each season (spring: Δ, autumn: ♦) against the species’ “lean” body mass, as given in the Handbook of the Birds of the World. Body mass values are presented in species-specific colours (Table 1). The blue dotted line shows the predicted relationship between the total speed of migration and body mass as detailed in eq. (7) by Hedenström [20]. The red dotted line (spring) and orange dotted line (autumn) indicate the observed effect of body mass on daily migration speed for 102 species of North American migratory birds that use powered flight, after La Sorte et al. [21]. Please consider that the approach used by these authors likely underestimated individual-based migration speeds and that in my approach, high total speeds of migration are likely biased due to missing the first migratory fuelling period and by low total speeds of migration obtained by underestimating the total migration distance. **b** Total speed of migration per season (spring: *n* = 32; autumn: *n* = 34) presented in boxplots (boxes present 5, 25, 50, 75, and 95% percentiles and outliners as dots). The Y-axes show the log10-transformed values of the total speed of migration

I found no effect of “lean” body mass (95% CI: − 0.15 – 0.41 km/g) on the total speed of migration (Fig. 5a). The latter parameter was significantly higher in spring than in autumn (95% CI: 0.11–0.26 km, the reference category for season was spring; both numeric variables were log10-transformed) (Fig. 5b).

## Discussion

The generality with which the start of migration correlated positively with arrival timing within and between species suggests that this is the general biological mechanism regulating arrival timing but not the total migration distance (Figs. 2 and 3). Through comprehensive analyses, this study further quantified how variation in the start of migration and the total migration distance may influence arrival timing at the migratory destination during spring and autumn (Table 2, Fig. 2) if the within- and between-species effects quantified by the models (Table 2) realistically represent the effects acting at the individual level. If appropriate, these findings may allow season-specific estimates of how variation in one migratory trait quantitatively alters arrival timing. Putting this into the ecological context of advanced arrival timing is an important step towards understanding the mechanisms whereby birds could potentially adjust the timing of their annual cycle in response to anthropogenic changes in the climate and environment. The generality with which the start of migration potentially explains advanced/delayed phenologies suggests that regulating arrival timing by variation in the start of migration is an important biological mechanism, cf. Ouwehand & Both [13]. At the same time, the suggested less-than-proportional advancement and the proposed limited effect of the total migration distance revealed a biologically significant contribution of the total speed of migration to arrival timing. How the start of migration, total migration distance, and total speed of migration are quantitatively related to arrival timing seems to be species specific (Fig. 3). In accordance with previous observations [18, 19], the total speed of migration increased with the total migration distance (Fig. 4). The total speed of migration was, however, not related to the species’ body mass (Fig. 5).

### Start of migration

There was a strong within- and between-species pattern in which an early start of migration translated to an early arrival at the migratory destination (Figs. 2 and 3). The magnitude of these species-specific effects varied considerably but was below one (Fig. 3), suggesting that the biological significance of the three migratory traits regulating arrival timing considered here differs between species. Reliable quantification of the delay of how much later an individual would arrive at the migratory destination compared to conspecifics starting migration one day earlier was, however, hampered by the small sample size for many species. Furthermore, we do not know to what extent the between-individual effects correctly reflect the within-individual effects.

Laboratory studies have demonstrated that the start of migration is subject to circannual and circadian control [80, 88] and is an inherited trait [89]. Under free-flying conditions, birds have been shown to flexibly adjust the start of migration to current environmental conditions, probably within their endogenously controlled reaction norm, with birds in better body condition advancing the start [29, 90]. In barn swallows, variation in the normalized difference vegetation index at their probable wintering grounds explained a within-individual difference of 27 days in breeding area arrival timing [91]. For this difference to arise, the start of spring migration would have had to vary by 68 days, provided that the general within-species effect of the start of spring migration on arrival timing of 0.4 was reasonable (Figs. 2 and 3) and that no other migratory trait varied, cf. Ouwehand & Both [13]. While between-individual variation in the start of spring migration (e.g., ranging from 30 to more than 60 days in barn swallows [92]) seems to be sufficiently large to potentially account for the observed advance in arrival timing, the small amount of available data on the within-individual variation in this trait (e.g., for the wood thrush (*Hylocichla mustelina*) (5 ± 5 days, *n* = 11; [11]) and the great reed warbler (11 ± 6 days, *n* = 4; [35])) suggests that phenotypic plasticity is important at the level of the individual [25, 26, 33]. For the overall observed advancement in breeding area arrival timing of 0.2 to 1 days per year to occur (see background), songbirds would have had to accelerate the start of their spring migration by 0.5 to 2.5 days each year. Such changes are probably jointly caused by phenotypically advancing the start of migration [29] and by directional selection for an earlier start of spring migration (i.e., rapid microevolution) [6, 13, 26, 93, 94].

### Total migration distance

Since a longer total migration distance correlated positively with arrival timing (Fig. 2), overwintering closer to the breeding area could potentially advance breeding area arrival timing [95, 96]. The estimated effect of the total migration distance on arrival timing was, however, rather small (Table 2). The between-individual data of a species may not adequately capture how much an individual could theoretically advance its arrival timing by shifting its wintering ground closer to the breeding area because between-individual differences in bird quality, wintering habitat [29], and the environmental conditions encountered en route [97] may have a stronger effect on arrival timing than the migration distance itself [59]. Furthermore, underestimated distances and between-study differences in the accuracy and precision of these estimates (for both see Methods) may disguise the potential effect of the total migration distance on arrival timing. Nevertheless, a shift in the wintering ground as a result of either phenotypic plasticity or evolutionary processes represents a potential adjustment or adaption to advance breeding area arrival timing [95]. However, the poleward shift of wintering grounds [98, 99] is generally counter-balanced by a simultaneous poleward shift of the corresponding breeding areas [98, 100] such that the total migration distance does not necessarily change over time. Regardless of the cardinal direction of the shift, the associated altered photoperiod will probably affect the start of spring migration [80], though the direction of the effect [101, 102] and how this carries over to breeding area arrival timing remain ambiguous [103]. In summary, variation in the total migration distance is likely to play a less important role than the start of migration on the variation in arrival timing.

### Total speed of migration

The total speed of migration was not considered in the models of the variation in arrival timing for statistical reasons, and thus, its effect is not captured by the models. Since the data quality of all considered migratory traits was limited [70, 71], the remaining variation left unexplained by the corresponding models (Table 2) could not be entirely attributed to variation in the total speed of migration. Thus, we could not quantify the effect of the latter on the variation in arrival timing with this approach. Birds can speed up their migration by increasing ground speed [104] either via greater wind support [105] and/or a higher air speed [106]. This increase is, however, limited to the maximum range speed [V_mr_] or the speed associated with the maximum migration speed [V_mt_] depending on whether time, the energy costs of transport or the total energy cost are minimized [107, 108]. Birds can further speed up their migration by reducing flights costs [109] and/or increasing the rate of energy accumulation [110]. Time minimizers can additionally speed up migration by resuming migration when the instantaneous speed of migration drops below the expected speed of migration [110, 111] (e.g., large energy stores at departure are associated with a high speed of migration [112]); see also Nilsson et al. [113] for further information about the total speed of migration. The rate of energy accumulation strongly affects the total stopover duration and is an important factor shaping the total speed of migration [30, 108, 110]. However, the potential increase in the rate of energy accumulation is not unlimited because migrants will eventually be metabolically limited [114]. Thus, the flexibility in the total stopover duration due to variation in the rate of energy accumulation is probably insufficient to explain the observed arrival advancements by itself [6]. For example, in Dutch pied flycatchers, breeding area arrival timing is determined by their African departure timing but not by the total speed of migration [13].

It seems to be a general phenomenon in birds that the total speed of migration is commonly higher in spring than autumn (Fig. 5b) [19, 113, 115]. Since the rate of energy accumulation is far slower than the rate of energy expenditure during flight [30, 108], songbirds usually spend more time at stopovers than in migratory flights [116–118]. Faster migration in spring is therefore mainly caused by a shorter total stopover duration resulting from higher rates of energy accumulation than in autumn [113, 115]. To the best of my knowledge, there is currently no evidence of an endogenously controlled seasonal difference in birds’ motivation to fuel [119]. Hence, higher rates of energy accumulation in spring are probably due to higher food availability [120–122], lower food-based competition [123], more-favourable weather (especially important for insectivorous species) [124] and/or more daylight hours, allowing longer daily feeding periods [125], among other factors. These differences could result in generally shorter stopover durations in spring than in autumn.

The total speed of migration was positively correlated with the total migration distance within and between species (only in spring) (Table 3, Fig. 4a), as statistically expected and shown for portions of the seasonal migratory movements of different populations [21]. In contrast to other studies [21–23], body mass was not negatively correlated with the total speed of migration in the present study (Fig. 5a). The low variation in body mass observed in this study (6.3–127 g) in comparison with that reported in others ([21]: 2.5–636 g; [23]: 12–10,350 g; [22]: 50–750 g) may have accounted for this difference because the low speeds of relatively heavy non-songbird species were mainly responsible for the effect observed in these other studies. Moreover, speed was estimated differently. This study and that of Watanabe [23] considered the individual total speed of migration. Zhao et al. [22] considered the individual partial speed of migration. La Sorte et al. [21] considered portions of the seasonal migratory movements of different populations. In combination with the different included species, these differences resulted in different estimates ([21]: 10–65 km/day; [23]: 10–1440 km/day; [22]: 25–300 km/day, and this study: 24–419 km/day).

In addition to these general differences, estimating the total speed of migration via light-level geolocation presents some inherent problems (see also Methods). Since this approach does not track daily fine-scale movements but provides “inexact” approximations of the general migratory route [70, 71], speed is generally underestimated. Furthermore, if the first migratory fuelling period (i.e., when accumulation of energy and size changes in different organs take place) occurs in close vicinity to the breeding area/wintering ground, then its duration cannot be captured. This biases the expected flight:stopover ratio towards less stopover time and, consequently, overestimates the total speed of migration. To the best of my knowledge, we so far lack detailed information about the first migratory fuelling period [126, 127] of songbirds in the wild before they actually start migration, but see Rubolini et al. [128] for the first migratory fuelling at a roost site. Since most songbird species probably encounter favourable feeding habitats along their migration route on a regular basis, the drive to accumulate large energy stores and extensively build up muscles before departure is generally less pronounced than in waders, for example. In the latter group, the occurrence of the first migratory fuelling period and extensive muscle development before the first migratory flight is a common phenomenon [129, 130] because these species often migrate over long distances to reach the next favourable stopover area, e.g., [72, 131]. Zhao et al. [22, 132] minimized this issue in waders by estimating the partial speed of migration. This approach starts with departure from the first stopover and not the initial migratory starting point. For waders, this approach is especially useful because they experience high energy depletion during long non-stop flight bouts to the first stopover, where they also fuel for a relatively long time so that they have to perform only a few flight bouts and stopovers to reach the migratory destination [22, 132]. Thus, missing the first of the very few fuelling periods leads to significant overestimation of the total speed of migration in waders. Songbirds, in contrast, follow a stop-and-go migration strategy with alternating cycles of relatively little energy accumulation for a few days and daily migratory movements [30]. The total number of flight bouts and fuelling periods is therefore much greater than in waders, so the flight:stopover ratio is more robust to missing a single short fuelling period. Nevertheless, between- and within-species-specific as well as seasonal differences in the duration of the first migratory fuelling period, potentially affecting the amount of energy accumulated before the first migratory flight, may yield different total speeds of migration [30, 112]. These differences may thus affect the between- and within-species slope between the start of migration and arrival timing, but see also La Sorte et al. [21]. Underestimating the total migration distance and total duration of migration to unknown degrees increases variation in the total speed of migration, which will decrease the statistical power to identify a statistically significant negative effect of lean body mass on the variation in the total speed of migration. To overcome this problem, future tracking studies should identify the duration of the first migratory fuelling period and detail migratory movements spatially accurately enough that more precise information on the start of migration and the total migration distance will eventually yield more accurate estimates of the total speed of migration. It will be interesting to determine whether future data confirms or leads to rejection of my results.

Crucial figures underlying the theoretical prediction of how body mass affects the total speed of migration are the rate of energy accumulation and the rate of energy expenditure during flight [20]. Both rates may diverge more strongly from the assumed scaling relationship and may vary more intensely within and between species than formerly anticipated. If so, then this would be another reason why no effect of body mass was found (Fig. 5a).

## Conclusions

This study illustrates the high potential for the start of migration to affect variation in arrival timing if the within-species effects quantified here correctly characterize within-individual effects. Since the anthropogenic changes in the climate and environment at wintering grounds are unlikely to coincide with the changes in breeding areas [133], but see also Pancerasa et al. [134], it is not a phenotypic response alone but also an evolutionary shift in the start of spring migration that most likely explains the advanced breeding area arrival timing [6, 13]. Additionally, environmental conditions phenotypically affect the total speed of migration through an influence of varying feeding conditions at stopover sites on the total stopover duration [30, 77] and an influence of varying wind conditions on ground speed [31]. My approach of estimating the potential effect of the start of migration and the total migration distance on the variation in arrival timing and not considering the total speed of migration is a simplification because the cumulative effect of all three migratory traits naturally defines arrival timing.

Furthermore, there may be species-specific constraints that hamper the potential degree of adjustment/adaptation [135]. Since songbirds undergo a complete moult at their breeding area or wintering ground [136], if this is not suspended [137], any advancement in the start of migration may be limited by the completion of moulting. The timing of birds’ annual cycles may be further constrained by the nest-laying date [138], exogenous phenological events (e.g., predation [139]), and/or feeding conditions en route [32]. Such constraints are probably species specific and, thus, responsible for some variation in the biological significance of the migratory traits regulating arrival timing (Fig. 3). More longitudinal data on the individual temporal organization of annual cycles, including the identification of the first migratory fuelling period and a high spatiotemporal resolution of migration data, are required to robustly quantify these mechanisms. Then we can investigate the crucial ecological and evolutionary questions of whether phenotypic adjustments are sufficiently strong to explain the current temporal variation in the main annual cycle events or whether directional selection towards an advanced start of migration is indeed the driving force behind the changes in arrival timing.

## Additional files

Additional file 1: **Figure S1.** Relationships among the bird species as considered in this study. Data were downloaded from www.timetree.org. The Newick code including the phylogenetic data is made available. (PDF 557 kb)

Additional file 2: Full R code for all analyses. (PDF 1223 kb)

Additional file 3: All tracking data used for the analyses. (XLSX 60 kb)

## Data Availability

All tracking data used for this study are made available in the Additional file 3.
