# Supplementary material for: The start of migration correlates with arrival timing, and the total speed of migration increases with migration distance in migratory songbirds: a cross-continental analysis
Source: Mov Ecol. 2019 Aug 12;7:25. doi: 10.1186/s40462-019-0169-1 (PMC6689889; doi:10.1186/s40462-019-0169-1)
Supplement: Supplementary file 1 — Figure S1. Relationships among the bird species as considered in this study. Data were downloaded from www.timetree.org. The Newick code including the phylogenetic data is made available. (PDF 557 kb) [file 40462_2019_169_MOESM1_ESM.pdf]

Additional file 1

The start of migration correlates with arrival timing, and the total speed of migration increases with migration distance in migratory songbirds: a cross-continental analysis

Heiko Schmaljohann

Fig. S1. Relationships among bird species as considered in this study. Data were downloaded from www.timetree.org. The Newick code including the phylogenetic data is made available.

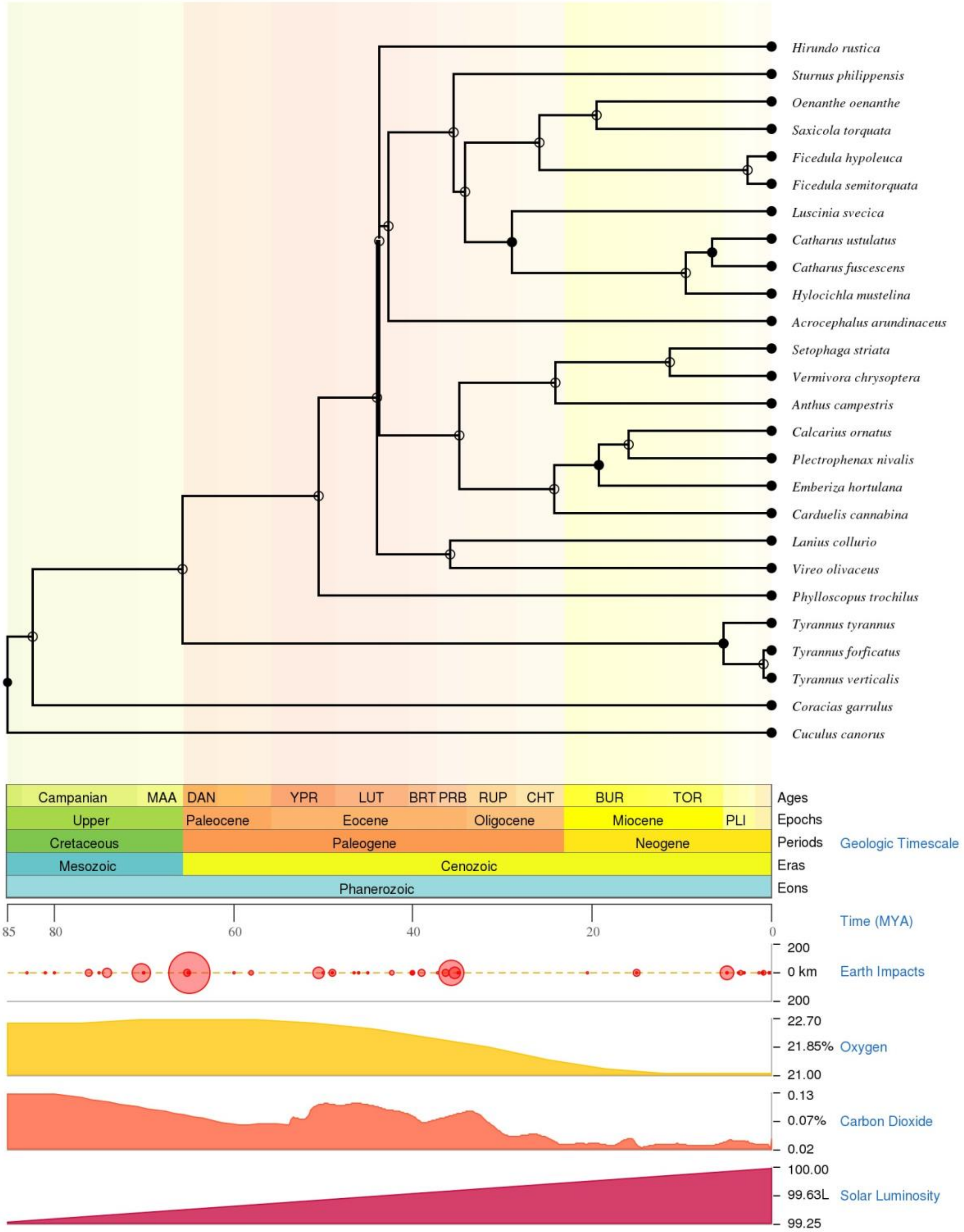

Newick file:

(Cuculus_canorus:85.20000000,(Coracias_garrulus:82.45491770,(((Tyrannus_verticalis:0.87126700,Tyrannus_forficatus:0.87126700)'14':4.49873300,Tyrannus_tyrannus:5.37000000)'13':60.32121089,(Phylloscopus_trochilus:50.54237727,(((Hirundo_rustica:43.70000000,((((((Catharus_fuscescens:6.69128500,Catharus_ustulatus:6.69128500)'11':2.88039600,Hylocichla_mustelina:9.57168100)'10':19.44060275,Luscinia_svecica:29.01228375)'19':5.18771625,((Ficedula_hypoleuca:2.72288567,Ficedula_semitorquata:2.72288567)'9':23.17544933,(Saxicola_torquata:19.45422150,Oenanthe_oenanthe:19.45422150)'22':6.44411350)'8':8.30166500)'6':1.24880857,Sturnus_philippensis:35.44880857)'30':7.28981874,Acrocephalus_arundinaceus:42.73862731)'29':0.96137269)'27':0.00000000,(((Setophaga_striata:11.36451000,Vermivora_chrysoptera:11.36451000)'35':12.70331500,Anthus_campestris:24.06782500)'43':10.73217500,(Carduelis_cannabina:24.27687333,((Plectrophenax_nivalis:15.90000000,Calcarius_ornatus:15.90000000)'42':3.30000000,Emberiza_hortulana:19.20000000)'40':5.07687333)'48':10.52312667)'51':8.90000000)'47':0.30000000,(Lanius_collurio:35.80000000,Vireo_olivaceus:35.80000000)'39':8.20000000)'56':6.54237727)'55':15.14883361)'61':16.76370681)'60':2.74508230);
