# Supplementary material for: The start of migration correlates with arrival timing, and the total speed of migration increases with migration distance in migratory songbirds: a cross-continental analysis
Source: Mov Ecol. 2019 Aug 12;7:25. doi: 10.1186/s40462-019-0169-1 (PMC6689889; doi:10.1186/s40462-019-0169-1)
Supplement: Supplementary file 2 — Full R code for all analyses. (PDF 1223 kb) [file 40462_2019_169_MOESM2_ESM.pdf]

Additional file 2: Full R code for all analyses

The start of migration correlates with arrival timing, and the total speed of migration increases with migration distance in migratory songbirds: a cross-continental analysis

Heiko Schmaljohann

Content:

1. General stuff
2. Adjusting and generating additional variables
3. Modelling variation in arrival timing at the breeding area
4. Modelling variation in arrival timing at the wintering ground
5. Modelling variation in total speed of migration via total migration distance
6. Modelling variation in total speed of migration via body mass
7. Modelling variation in total speed of migration via body mass and controlling for shared ancestry

1. General stuff

Libraries

```
library(arm)
library(blmeco)
library(lme4)
library(maptools)
library(MuMIn)
library(plyr)
library(shape)
library(usdm)
library(phytools)
library(nlme)
library(optimx)
```

Set time to GMT

```
Sys.setenv(tz="GMT")
```

Define and set work directory

```
wd <- "your work directory"
setwd(wd)
```

Read data

```
dat <- read.csv("individual data on arrival timing.csv", sep=",", header = T, dec = ".")
```

2. Adjusting and generating additional variables

```
# a) converting dates in Julian dates
# start_mig = date when migration was started
dat$start_mig.jd <- strptime(dat$start_mig, "%d/%m/%Y")$yday+1
# end_mig = date when migration was terminated
```

```
dat$end_mig.jd <- strptime(dat$end_mig, "%d/%m/%Y")$yday+1

# b) defining color per ID
t.levels <- levels(dat$ID)
t.col <- intpalette(c("olivedrab3", "orange", "blue", "yellow1",  "black"),#c("grey75", "olivedrab3", "orange", "lightgrey",  "blue", "yellow2"),
                numcol=length(t.levels))
dat$t.col <- t.col[as.numeric(dat$ID)]
```

3. Modelling variation in arrival timing at the breeding area

3.1. Adjusting data

```
# a) generating variables
# selecting spring data and excluding NA-values
s.dat <- dat[dat$season=="spr" & !is.na(dat$end_mig.jd) & !is.na(dat$start_mig.jd),]
# reset levels of factor "ID"
s.dat$ID <- factor(s.dat$ID)
# renaming variable (arr =  arrival timing)
s.dat$arr.spr.mig.jd <- s.dat$end_mig.jd
# scaling variables
s.dat$arr.spr.mig.jd_s <- scale(s.dat$arr.spr.mig.jd, center = FALSE)
s.dat$start_mig.jd_s <- scale(s.dat$start_mig.jd, center = FALSE)
s.dat$total.dist_s <- scale(s.dat$total.dist, center = FALSE)
s.dat$t.speed_s <- scale(s.dat$t.speed, center = FALSE)

# To distinguish between within- versus between-species effects, I followed the recommendations of van de Pol & Wright (2009, Animal Behaviour 77: 753-758).
# b)  within-species variation captured by within-species centering
for (i in levels(s.dat$ID))
{ s.dat$start_mig.jd_s_ws.center[s.dat$ID == i] <- scale(s.dat$start_mig.jd_s[s.dat$ID == i], scale = FALSE)
  s.dat$total.dist_s_ws.center[s.dat$ID == i] <- scale(s.dat$total.dist_s[s.dat$ID == i], scale = FALSE)}
# Centering around species mean effectively eliminates any between-species variation. This provides two new fixed effects expressing only their within-species variation (van del Pol & Wright 2009).

# c) between-species variation captured by the species's mean
for (i in levels(s.dat$ID))
{ s.dat$start_mig.jd_s_s.mean[s.dat$ID == i] <- mean(s.dat$start_mig.jd_s[s.dat$ID == i])
  s.dat$total.dist_s_s.mean[s.dat$ID == i] <- mean(s.dat$total.dist_s[s.dat$ID == i])}

# d) assessing collinearity of explanatory variables for each species
# species with collinearity >3, cf. Zuur et al. (2010, Methods in Ecology and Evolution 1: 3-14) are given
# only species with more than three individuals are considered
species.coll.spr <- c()
n <- 0
for(i in levels(s.dat$ID))
{tmp <- vif(s.dat[s.dat$ID == i,c("start_mig.jd", "total.dist")])[1,2]
  if (tmp>3)
   {n <- n+1
```

```
    print(c(i, round(tmp,2)))
    species.coll.spr[n] <- i # captures species, in which explanatory variables are collinear
   }
   else
  if (nrow(s.dat[s.dat$ID == i,c("start_mig.jd", "total.dist")])==1)
   {n <- n+1
    print(c(i, round(tmp,2)))
    species.coll.spr[n] <- i # captures species, in which explanatory variables are collinear
   }
     else
     (next)
}
```

```
## [1] "blackpoll warbler" "8.25"
## [1] "chestnutcollared longspur" "4.9"
## [1] "common cuckoo" "6.35"
## [1] "pied flycatcher" "4.78"
## [1] "red-spotted bluethroat" "Inf"
## [1] "western kingbird" "Inf"
```

```
# number of species with collinear explanatory variables
length(species.coll.spr)
```

```
## [1] 6
```

3.2. Modelling variation in arrival timing at the breeding area

```
# a) modelling
mod <- lmer(arr.spr.mig.jd_s ~ start_mig.jd_s_ws.center +
                    total.dist_s_ws.center +
                    start_mig.jd_s_s.mean +
                    total.dist_s_s.mean +
                    (start_mig.jd_s_ws.center|ID),
                    s.dat,
                    REML = FALSE,
                    lmerControl(optimizer ='optimx', optCtrl=list(method='L-BFGS-B')),
                    subset = !(s.dat$ID%in%species.coll.spr))



# This model was initially run with the two-way interaction "start_mig.jd_s_ws.center:total.dist_s_ws.cen
ter". The corresponding 95% CrI (-0.38, 0.36) included zero and was therefore removed.

# b) assessing model's assumptions

# compare plot to simulated data
par(mar=rep(4,4))
compareqqnorm(mod)
```

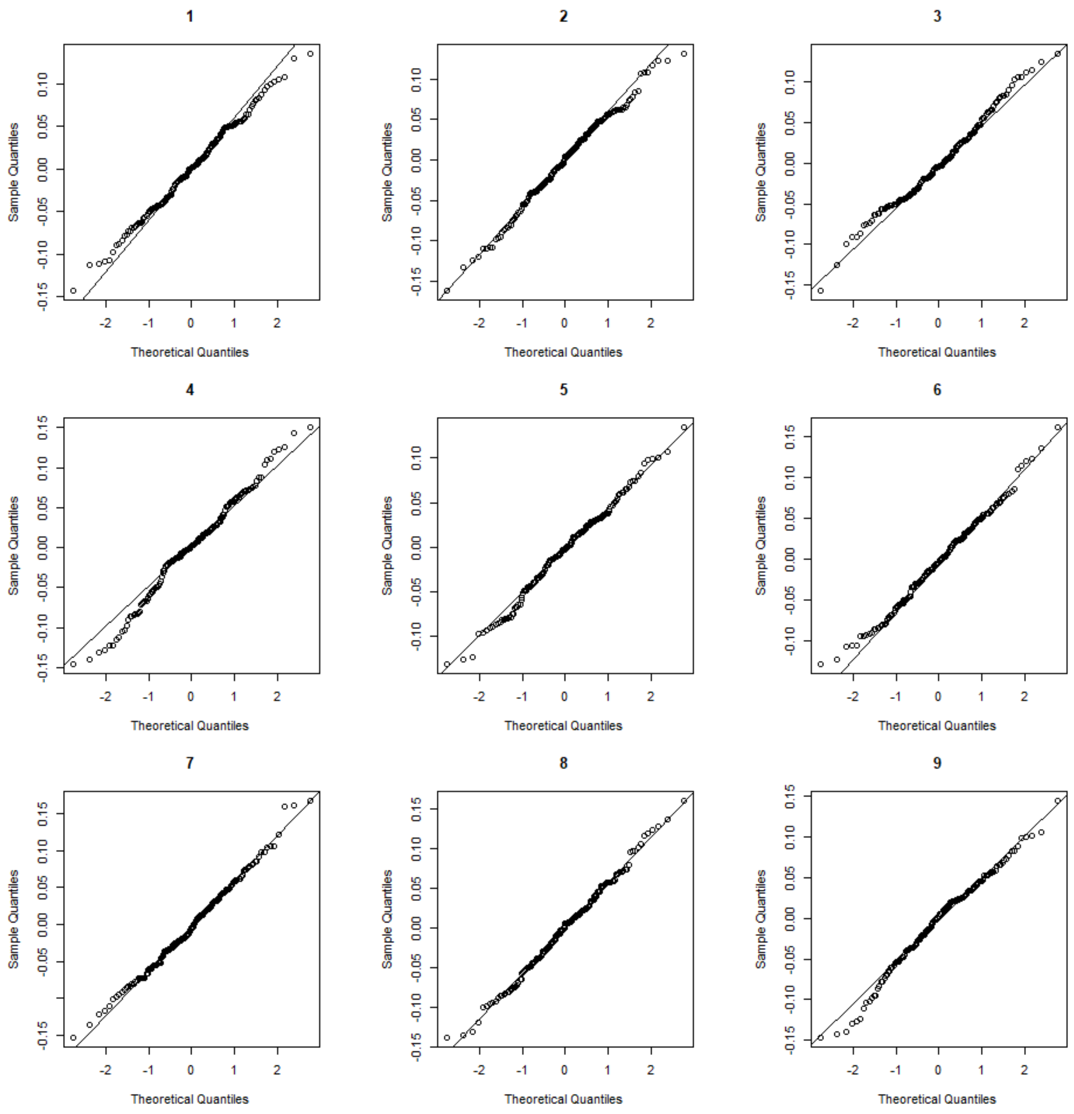


```
## [1] 3

# --> no violation

# autocorrelation
par(mfrow=c(1,2), mar=rep(4,4))
acf(resid(mod))
acf(resid(mod),type="p")
```

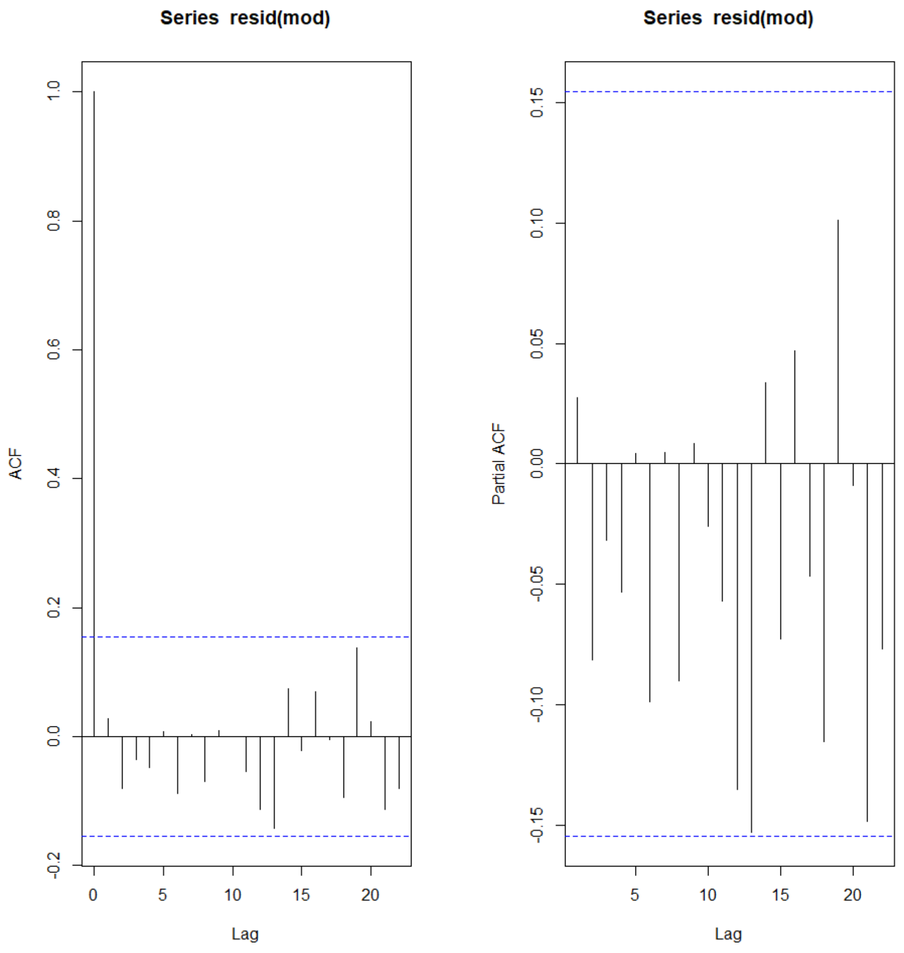


```
# --> no violation

# residual plots
t.cex <- 1
par(mfrow=c(3,2), mar=rep(4,4), mgp=c(3,1,0))
# Tukey-Ascombe plot: Residuals over predicted values
scatter.smooth(fitted(mod), resid(mod), main="Tukey-Anscombe Plot", cex=t.cex)
abline(h=0, lty=2) # residuals vs. fitted

# normal QQ plot of the residuals
qqnorm(resid(mod), main="Normal QQ plot, residuals", cex.main=t.cex) # qq of residuals
qqline(resid(mod))

# square-root of the absolute values of the residuals versus fitted values
scatter.smooth(fitted(mod), sqrt(abs(resid(mod))), main = "Scale-Location") # res. var vs. fitted

# several QQ plots of random slopes and intercepts
qqnorm(ranef(mod)$ID[,1], main="")
qqline(ranef(mod)$ID[,1])
title(main="Normal QQ plot of the random intercepts \nper species (start_mig.jd_s_ws.center)",
   cex=t.cex)
```

```
qqnorm(ranef(mod)$ID[,2], main="")
qqline(ranef(mod)$ID[,2])
title(main="Normal QQ plot of the random slopes \nper species (start_mig.jd_s_ws.center)",
    cex=t.cex)
plot.new(); plot.new()
```

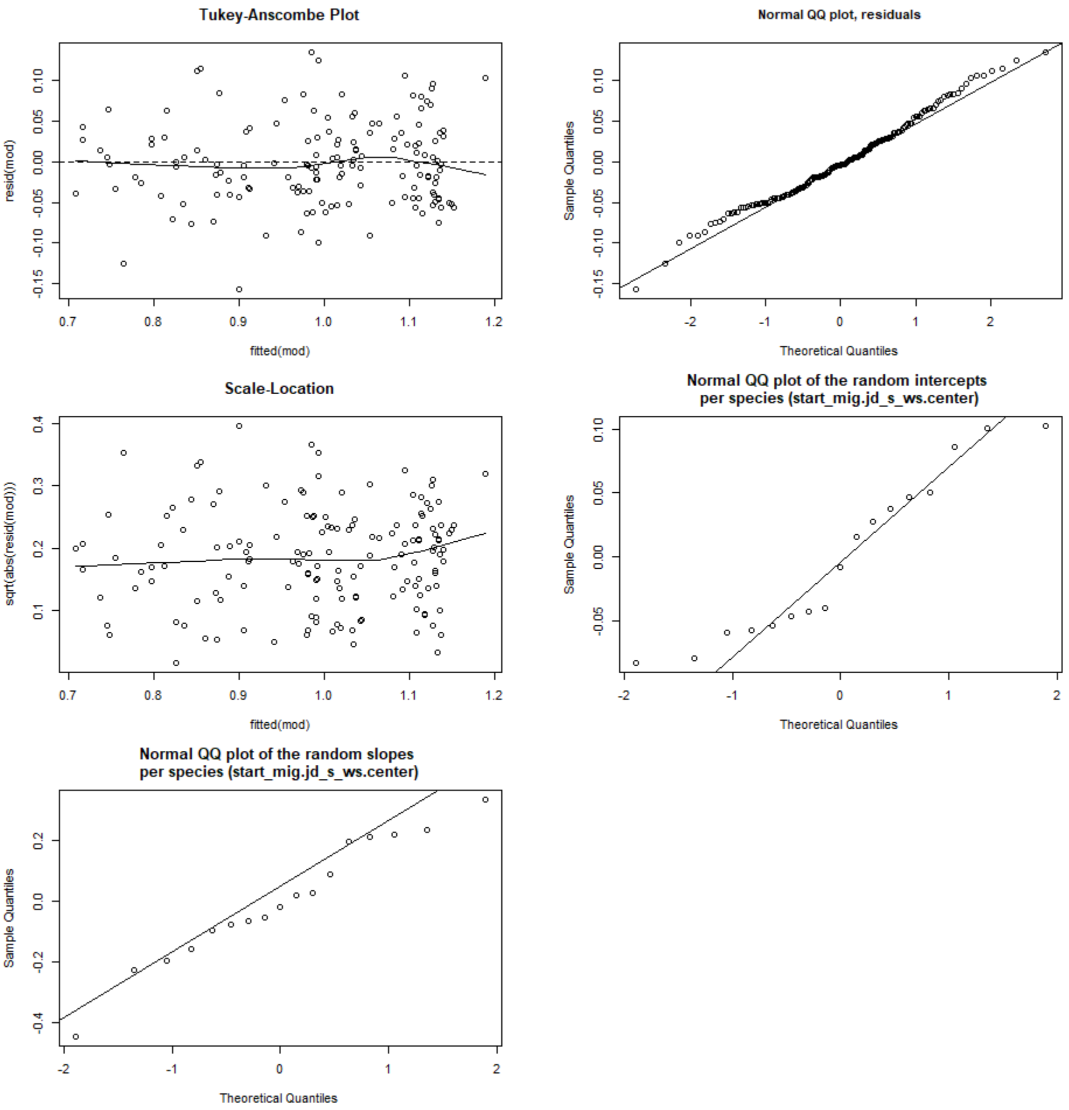


```
# --> no violation

# c) drawing conclusions
nsim <- 5000
set.seed(0470)    # specify the seed (starting value for random generator)
bsim <- sim(mod, n.sim=nsim)
colnames(bsim@fixef) <- names(fixef(mod))
r.fixef <- round(apply(bsim@fixef, 2, quantile, prob=c(0.025,0.5,0.975)),2)
r.fixef
```

```
##      (Intercept) start_mig.jd_s_ws.center total.dist_s_ws.center
## 2.5%        0.36                     0.12                   0.06
## 50%         0.51                     0.28                   0.10
## 97.5%       0.65                     0.43                   0.14
##      start_mig.jd_s_s.mean total.dist_s_s.mean
## 2.5%                  0.27                0.02
## 50%                   0.40                0.10
## 97.5%                 0.53                0.17

# number of individuals considered
length(mod@resp$y)

## [1] 161

# number of species considered
length(levels(mod@frame$ID))

## [1] 17

# model output
mod

## Linear mixed model fit by maximum likelihood  ['lmerMod']
## Formula:
## arr.spr.mig.jd_s ~ start_mig.jd_s_ws.center + total.dist_s_ws.center +
##     start_mig.jd_s_s.mean + total.dist_s_s.mean + (start_mig.jd_s_ws.center |
##     ID)
##    Data: s.dat
##  Subset: !(s.dat$ID %in% species.coll.spr)
##       AIC       BIC    logLik  deviance  df.resid
## -403.7839 -376.0513  210.8919 -421.7839       152
## Random effects:
##  Groups   Name                     Std.Dev. Corr
##  ID       (Intercept)              0.06430
##           start_mig.jd_s_ws.center 0.24447  -0.63
##  Residual                          0.05541
## Number of obs: 161, groups:  ID, 17
## Fixed Effects:
##             (Intercept)  start_mig.jd_s_ws.center
##                 0.50841                   0.28151
##   total.dist_s_ws.center     start_mig.jd_s_s.mean
##                 0.10208                   0.39931
##      total.dist_s_s.mean
##                 0.09719

# marginal and conditional R-square
r.squaredGLMM(mod)

## Warning: 'r.squaredGLMM' now calculates a revised statistic. See the help
## page.
```

```
##          R2m       R2c
## [1,] 0.5361183 0.8199574
```

Back-transformed effects

```
# 1. within-species
  # a) start of migration
  round(fixef(mod)[2] * (attr(s.dat$arr.spr.mig.jd_s, 'scaled:scale')/attr(s.dat$start_mig.jd_s, 'scaled:scal
e')),2)

## start_mig.jd_s_ws.center
##                     0.39

  # b) total migration distance per 1,000 km
  round(fixef(mod)[3] * (attr(s.dat$arr.spr.mig.jd_s, 'scaled:scale')/attr(s.dat$total.dist_s, 'scaled:scale')),
4)*1000

## total.dist_s_ws.center
##                    2.3

# 2. between-species effect
  # a) start of migration
  round(fixef(mod)[4] * (attr(s.dat$arr.spr.mig.jd_s, 'scaled:scale')/attr(s.dat$start_mig.jd_s, 'scaled:scal
e')),2)

## start_mig.jd_s_s.mean
##                 0.56

  # b) total migration distance per 1,000 km
  round(fixef(mod)[5] * (attr(s.dat$arr.spr.mig.jd_s, 'scaled:scale')/attr(s.dat$total.dist_s, 'scaled:scale')),
4)*1000

## total.dist_s_s.mean
##                2.1
```

4. Modelling variation in arrival timing at the wintering ground

4.1. Adjusting data

```
# a) generating variables
# selecting spring data and excluding NA-values
a.dat <- dat[dat$season=="aut" & !is.na(dat$end_mig.jd) & !is.na(dat$start_mig.jd),]
# reset levels of factor "ID"
a.dat$ID <- factor(a.dat$ID)
# renaming variable (arr =  arrival timing)
a.dat$arr.aut.mig.jd <- a.dat$end_mig.jd
# scaling variables
a.dat$arr.aut.mig.jd_s <- scale(a.dat$arr.aut.mig.jd, center = FALSE)
a.dat$start_mig.jd_s <- scale(a.dat$start_mig.jd, center = FALSE)
a.dat$total.dist_s <- scale(a.dat$total.dist, center = FALSE)
a.dat$t.speed_s <- scale(a.dat$t.speed, center = FALSE)
```

```
# To distinguish between within- versus between-species effects, I followed the recommendations of van de Pol & Wright (2009, Animal Behaviour 77: 753-758).
# b)  within-species variation captured by within-species centering
for (i in levels(a.dat$ID))
{ a.dat$start_mig.jd_s_ws.center[a.dat$ID == i] <- scale(a.dat$start_mig.jd_s[a.dat$ID == i], scale = FALSE
)
  a.dat$total.dist_s_ws.center[a.dat$ID == i] <- scale(a.dat$total.dist_s[a.dat$ID == i], scale = FALSE)}
# Centering around species mean effectively eliminates any between-species variation. This provides two new fixed effects expressing only their within-species variation (van del Pol & Wright 2009).

# c) between-species variation captured by the species's mean
for (i in levels(a.dat$ID))
{ a.dat$start_mig.jd_s_s.mean[a.dat$ID == i] <- mean(a.dat$start_mig.jd_s[a.dat$ID == i])
  a.dat$total.dist_s_s.mean[a.dat$ID == i] <- mean(a.dat$total.dist_s[a.dat$ID == i])}

# d) assessing collinearity of explanatory variables for each species
# species with collinearity >3, cf. Zuur et al. (2010, Methods in Ecology and Evolution 1: 3-14) are given
# only species with more than three individuals are considered
species.coll.aut <- c()
n <- 0
for(i in levels(a.dat$ID))
{tmp <- vif(a.dat[a.dat$ID == i,c("start_mig.jd", "total.dist")])[1,2]
  if (tmp>3)
    {n <- n+1
     print(c(i, round(tmp,2)))
     species.coll.aut[n] <- i # captures species, in which explanatory variables are collinear
    }
    else
  if (nrow(a.dat[a.dat$ID == i,c("start_mig.jd", "total.dist")])==1)
    {n <- n+1
     print(c(i, round(tmp,2)))
     species.coll.aut[n] <- i # captures species, in which explanatory variables are collinear
    }
     else
     (next)
}

## [1] "red-eyed vireo" "1"
## [1] "red-spotted bluethroat" "16.4"
## [1] "scissor-tailed flycatcher" "138.66"

## Warning in summary.lm(lm(y[, i] ~ ., data = y[-i])): essentially perfect
## fit: summary may be unreliable

## [1] "veery" "3.43"

# number of species with collinear explanatory variables
length(species.coll.aut)
```

```
## [1] 4
```

4.2. Modelling variation in arrival timing at the wintering ground

```
# a) modelling
mod <- lmer(arr.aut.mig.jd_s ~ start_mig.jd_s_ws.center +
                    total.dist_s_ws.center +
                    start_mig.jd_s_s.mean +
                    total.dist_s_s.mean +
                    (start_mig.jd_s_ws.center|ID),
                    a.dat,
                    REML = TRUE,
                    subset = !(a.dat$ID%in%species.coll.aut))

# This model was initially run with the two-way interaction "start_mig.jd_s_ws.center:total.dist_s_ws.cen
ter". The corresponding 95% CrI (-0.49, 0.35) included zero and was therefore removed.

# b) assessing model's assumptions

# compare plot to simulated data
par(mar=rep(4,4))
compareqqnorm(mod)
```

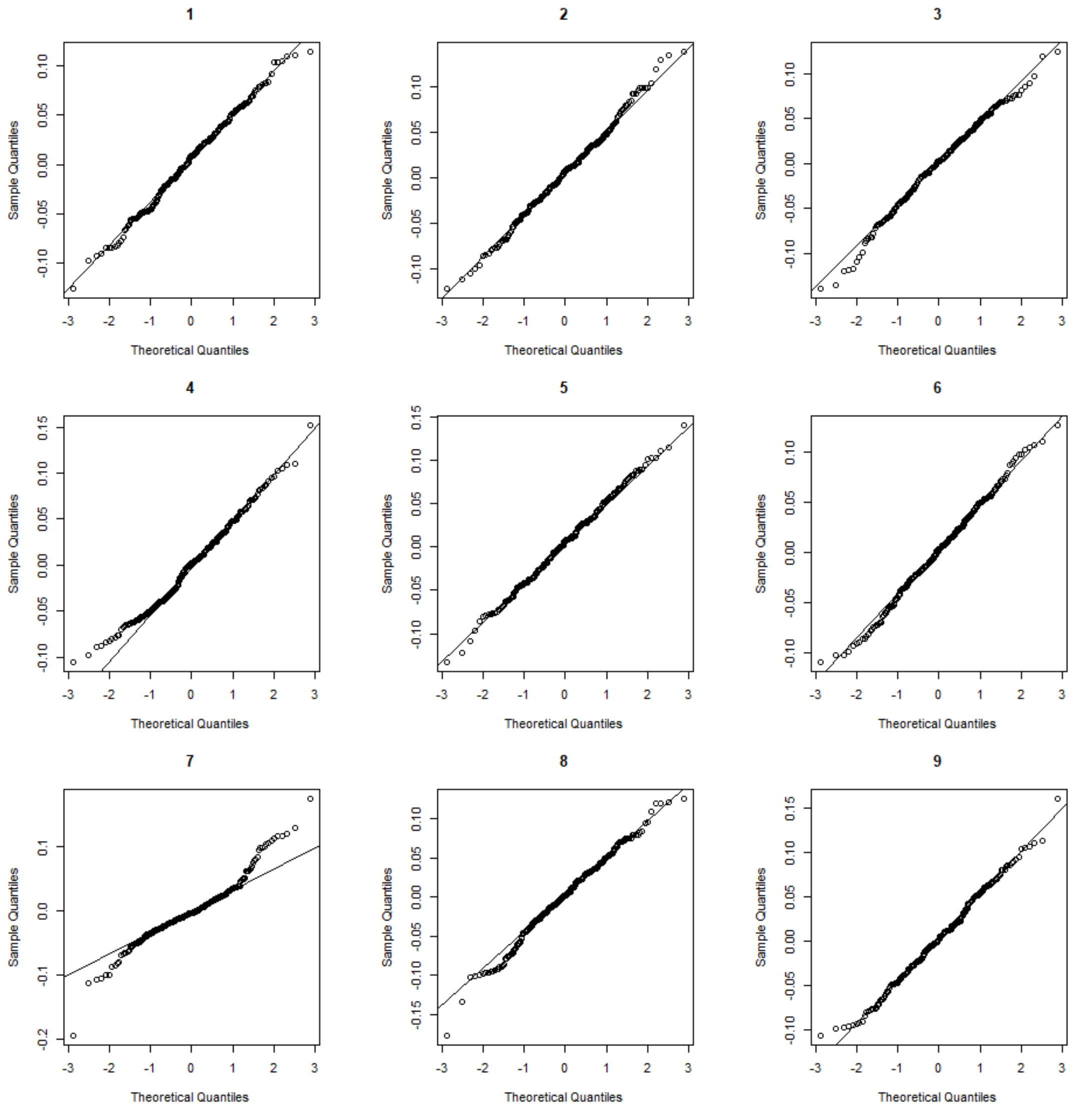


```
## [1] 7

# --> no violation

# autocorrelation
par(mfrow=c(1,2), mar=rep(4,4))
acf(resid(mod))
acf(resid(mod),type="p")
```

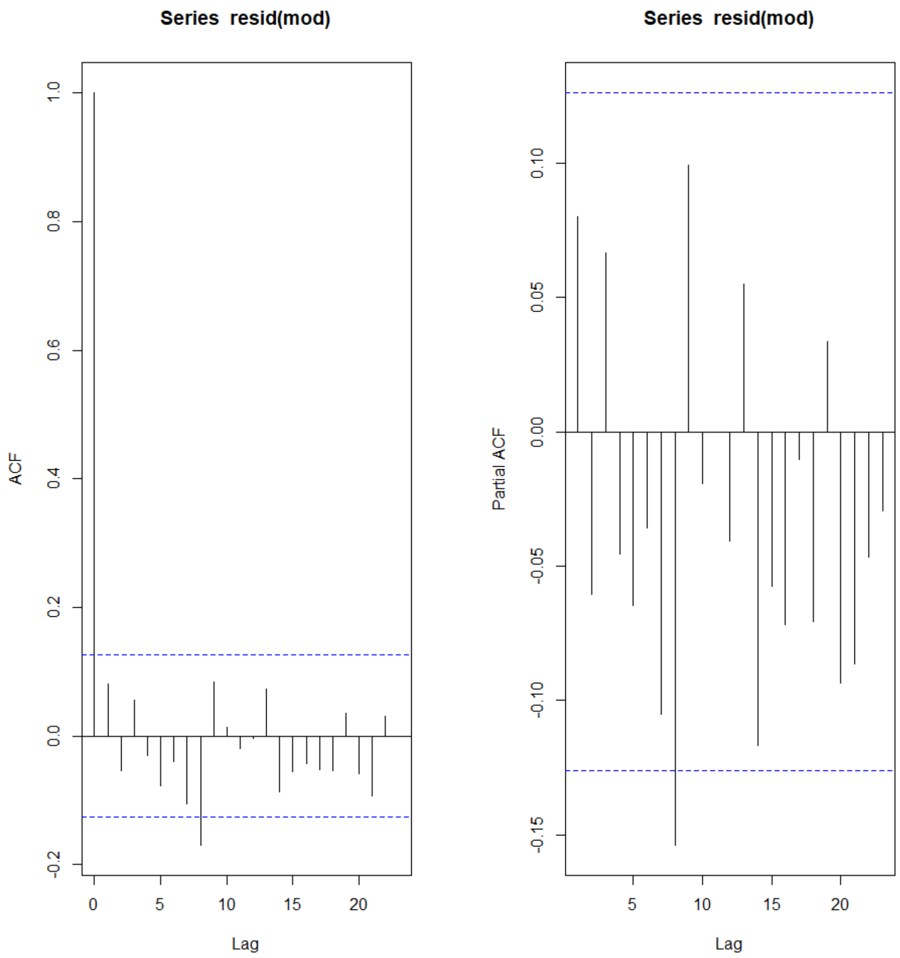


```
# --> no violation

# residual plots
t.cex <- 1
par(mfrow=c(3,2), mar=rep(4,4), mgp=c(3,1,0))
# Tukey-Ascombe plot: Residuals over predicted values
scatter.smooth(fitted(mod), resid(mod), main="Tukey-Anscombe Plot", cex=t.cex)
abline(h=0, lty=2) # residuals vs. fitted

# normal QQ plot of the residuals
qqnorm(resid(mod), main="Normal QQ plot, residuals", cex.main=t.cex) # qq of residuals
qqline(resid(mod))

# square-root of the absolute values of the residuals versus fitted values
scatter.smooth(fitted(mod), sqrt(abs(resid(mod))), main = "Scale-Location") # res. var vs. fitted

# several QQ plots of random slopes and intercepts
qqnorm(ranef(mod)$ID[,1], main="")
qqline(ranef(mod)$ID[,1])
title(main="Normal QQ plot of the random intercepts \nper species (start_mig.jd_s_ws.center)",
```

```r
    cex=t.cex)

qqnorm(ranef(mod)$ID[,2], main="")
qqline(ranef(mod)$ID[,2])
title(main="Normal QQ plot of the random slopes \nper species (start_mig.jd_s_ws.center)",
    cex=t.cex)
plot.new(); plot.new()
```

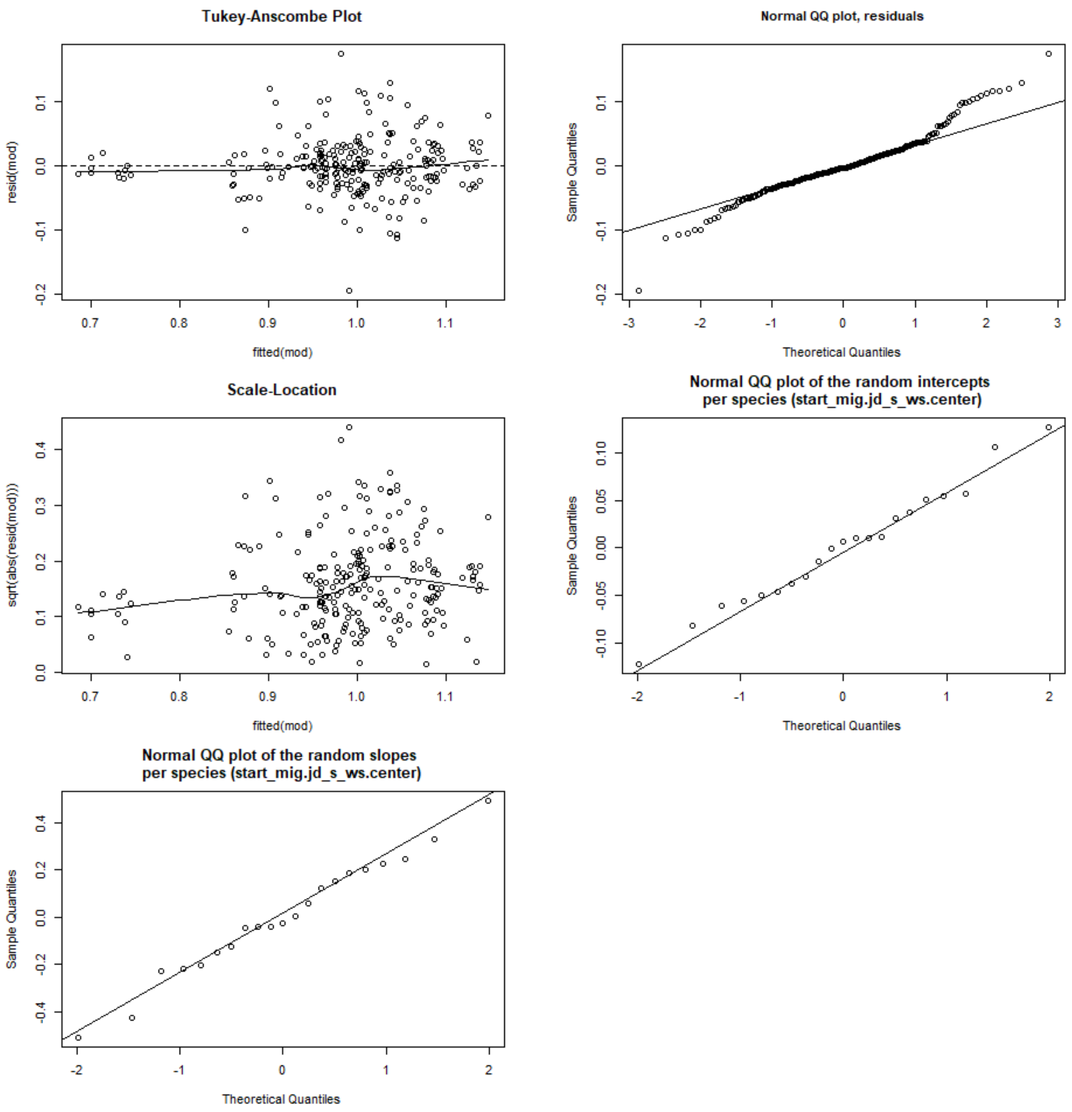


```r
# --> no violation

# c) drawing conclusions
nsim <- 5000
set.seed(0470)    # specify the seed (starting value for random generator)
bsim <- sim(mod, n.sim=nsim)
colnames(bsim@fixef) <- names(fixef(mod))
r.fixef <- round(apply(bsim@fixef, 2, quantile, prob=c(0.025,0.5,0.975)),2)
r.fixef
```

```
##       (Intercept) start_mig.jd_s_ws.center total.dist_s_ws.center
## 2.5%        0.04                   0.16                 -0.01
## 50%         0.29                   0.33                  0.02
## 97.5%       0.54                   0.49                  0.06
##       start_mig.jd_s_s.mean total.dist_s_s.mean
## 2.5%                  0.39                0.05
## 50%                   0.62                0.10
## 97.5%                 0.85                0.16

# number of individuals considered
length(mod@resp$y)

## [1] 241

# number of species considered
length(levels(mod@frame$ID))

## [1] 21

# model output
mod

## Linear mixed model fit by REML ['lmerMod']
## Formula:
## arr.aut.mig.jd_s ~ start_mig.jd_s_ws.center + total.dist_s_ws.center +
##     start_mig.jd_s_s.mean + total.dist_s_s.mean + (start_mig.jd_s_ws.center |
##     ID)
##    Data: a.dat
##  Subset: !(a.dat$ID %in% species.coll.aut)
## REML criterion at convergence: -700.2712
## Random effects:
##  Groups   Name                     Std.Dev. Corr
##  ID       (Intercept)              0.06485
##           start_mig.jd_s_ws.center 0.26155  -1.00
##  Residual                          0.04768
## Number of obs: 241, groups:  ID, 21
## Fixed Effects:
##             (Intercept)  start_mig.jd_s_ws.center
##                 0.28837                   0.32584
##   total.dist_s_ws.center     start_mig.jd_s_s.mean
##                 0.02497                   0.61802
##      total.dist_s_s.mean
##                 0.10283

# marginal and conditional R-square
r.squaredGLMM(mod)

##            R2m       R2c
## [1,] 0.3941459 0.7946291
```

Back-transformed effects

```
# 1. within-species
  # a) start of migration
  round(fixef(mod)[2] * (attr(a.dat$arr.aut.mig.jd_s, 'scaled:scale')/attr(a.dat$start_mig.jd_s, 'scaled:scal
e')),2)

## start_mig.jd_s_ws.center
##                      0.41

  # b) total migration distance per 1,000 km
  round(fixef(mod)[3] * (attr(a.dat$arr.aut.mig.jd_s, 'scaled:scale')/attr(a.dat$total.dist_s, 'scaled:scale'))
,4)*1000

## total.dist_s_ws.center
##                      1.2

# 2. between-species effect
  # a) start of migration
  round(fixef(mod)[4] * (attr(a.dat$arr.aut.mig.jd_s, 'scaled:scale')/attr(a.dat$start_mig.jd_s, 'scaled:scal
e')),2)

## start_mig.jd_s_s.mean
##                   0.77

  # b) total migration distance per 1,000 km
  round(fixef(mod)[5] * (attr(a.dat$arr.aut.mig.jd_s, 'scaled:scale')/attr(a.dat$total.dist_s, 'scaled:scale'))
,4)*1000

## total.dist_s_s.mean
##                 4.8
```

5. Modelling variation in total speed of migration via total migration distance

5.1. Adjusting data

```
# log10-transformation of dependent and independent variables and producing within- and between-spe
cies effects (see above for explanations and see van der Pol & Wright 2009, Anim Behav)

# a) spring data
s.dat$t.speed.log10 <- log10(s.dat$t.speed)
s.dat$total.dist.log10 <- log10(s.dat$total.dist)
for (i in levels(s.dat$ID))
s.dat$total.dist.log10_ws.center[s.dat$ID == i] <- scale(s.dat$total.dist.log10[s.dat$ID == i], center = TRU
E, scale = FALSE)
for (i in levels(s.dat$ID))
s.dat$total.dist.log10_s.mean[s.dat$ID == i] <- mean(s.dat$total.dist.log10[s.dat$ID == i])

# b) autumn data
a.dat$t.speed.log10 <- log10(a.dat$t.speed)
a.dat$total.dist.log10 <- log10(a.dat$total.dist)
for (i in levels(a.dat$ID))
```

```
a.dat$total.dist.log10_ws.center[a.dat$ID == i] <- scale(a.dat$total.dist.log10[a.dat$ID == i], center = TRU
E, scale = FALSE)
for (i in levels(a.dat$ID))
a.dat$total.dist.log10_s.mean[a.dat$ID == i] <- mean(a.dat$total.dist.log10[a.dat$ID == i])
```

5.2. Spring model

```
# a) modelling
mod <- lmer(t.speed.log10 ~  total.dist.log10_ws.center +
                        total.dist.log10_s.mean +
                        (total.dist.log10_ws.center|ID),
                        s.dat,
                        REML = TRUE)

# b) assessing model's assumptions

# compare plot to simulated data
par(mar=rep(4,4))
compareqqnorm(mod)
```

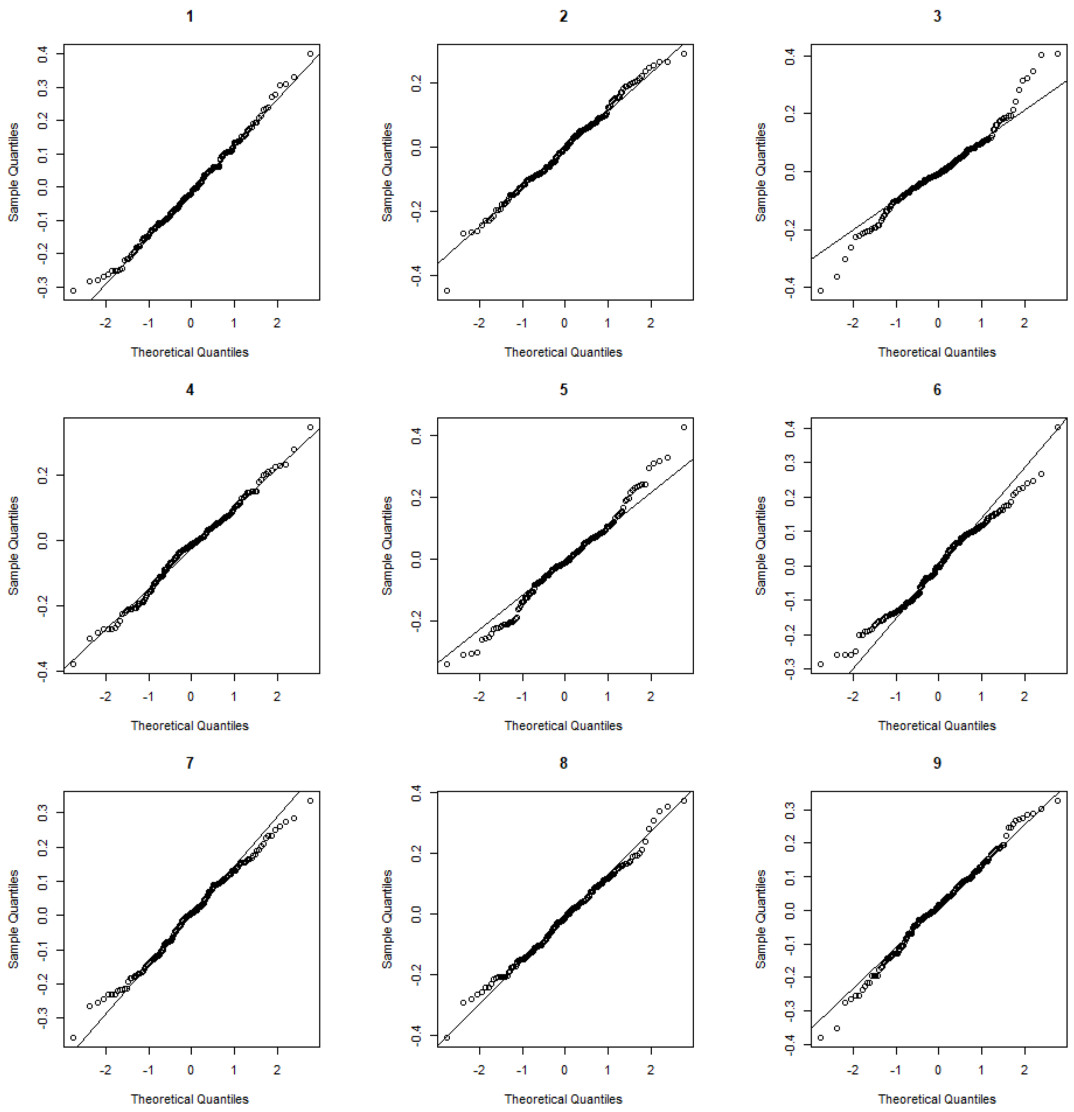


```
## [1] 3

# --> no violation

# autocorrelation
par(mfrow=c(1,2), mar=rep(4,4))
acf(resid(mod))
acf(resid(mod),type="p")
```

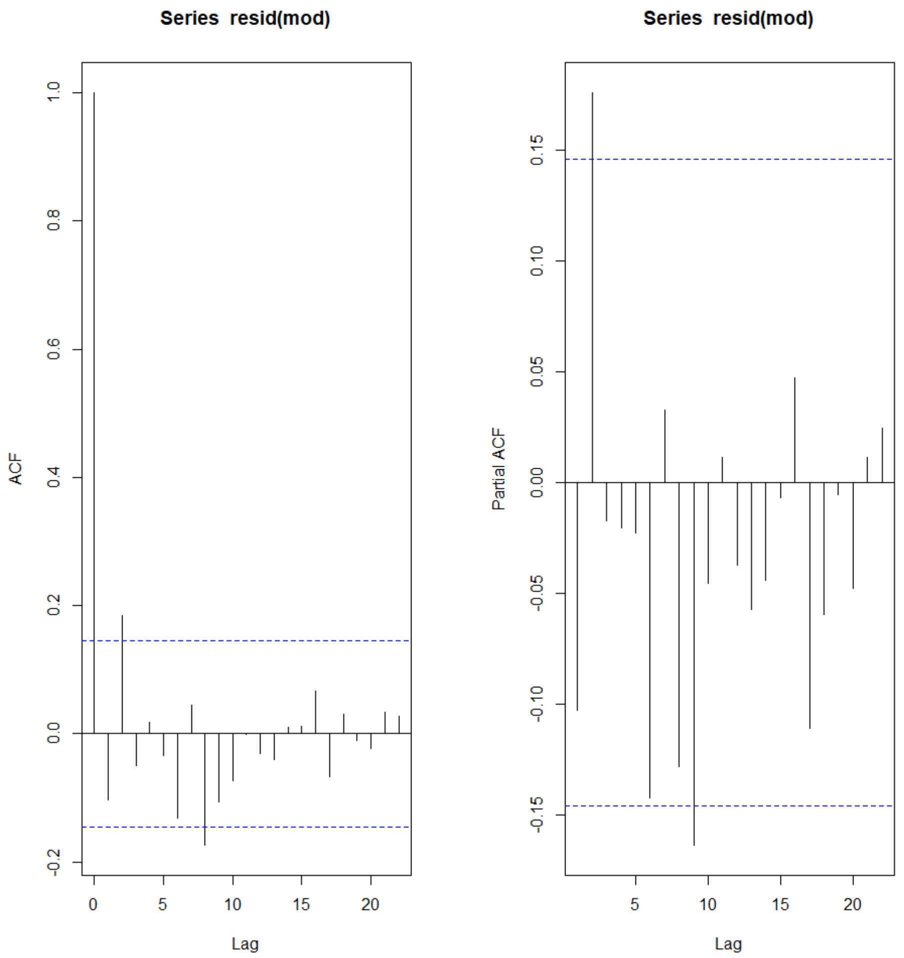


```
# --> no violation

# residual plots
t.cex <- 1
par(mfrow=c(2,3), mar=rep(4,4), mgp=c(3,1,0))
# Tukey-Ascombe plot: Residuals over predicted values
scatter.smooth(fitted(mod), resid(mod), main="Tukey-Anscombe Plot", cex=t.cex)
abline(h=0, lty=2) # residuals vs. fitted

# normal QQ plot of the residuals
qqnorm(resid(mod), main="Normal QQ plot, residuals", cex.main=t.cex) # qq of residuals
qqline(resid(mod))

# square-root of the absolute values of the residuals versus fitted values
scatter.smooth(fitted(mod), sqrt(abs(resid(mod))), main="Scale-Location") # res. var vs. fitted

# several QQ plots of random slope and intercept
qqnorm(ranef(mod)$ID[,2], main="")
qqline(ranef(mod)$ID[,2])
title(main="Normal QQ plot of the random slope \nper species  (total.dist.log10_s_ws.center)",
```

```
    cex=t.cex)

qqnorm(ranef(mod)$ID[,1], main="")
qqline(ranef(mod)$ID[,1])
title(main="Normal QQ plot of the random intercept \nper species (total.dist.log10_s_ws.center)",
    cex=t.cex)
plot.new()
```

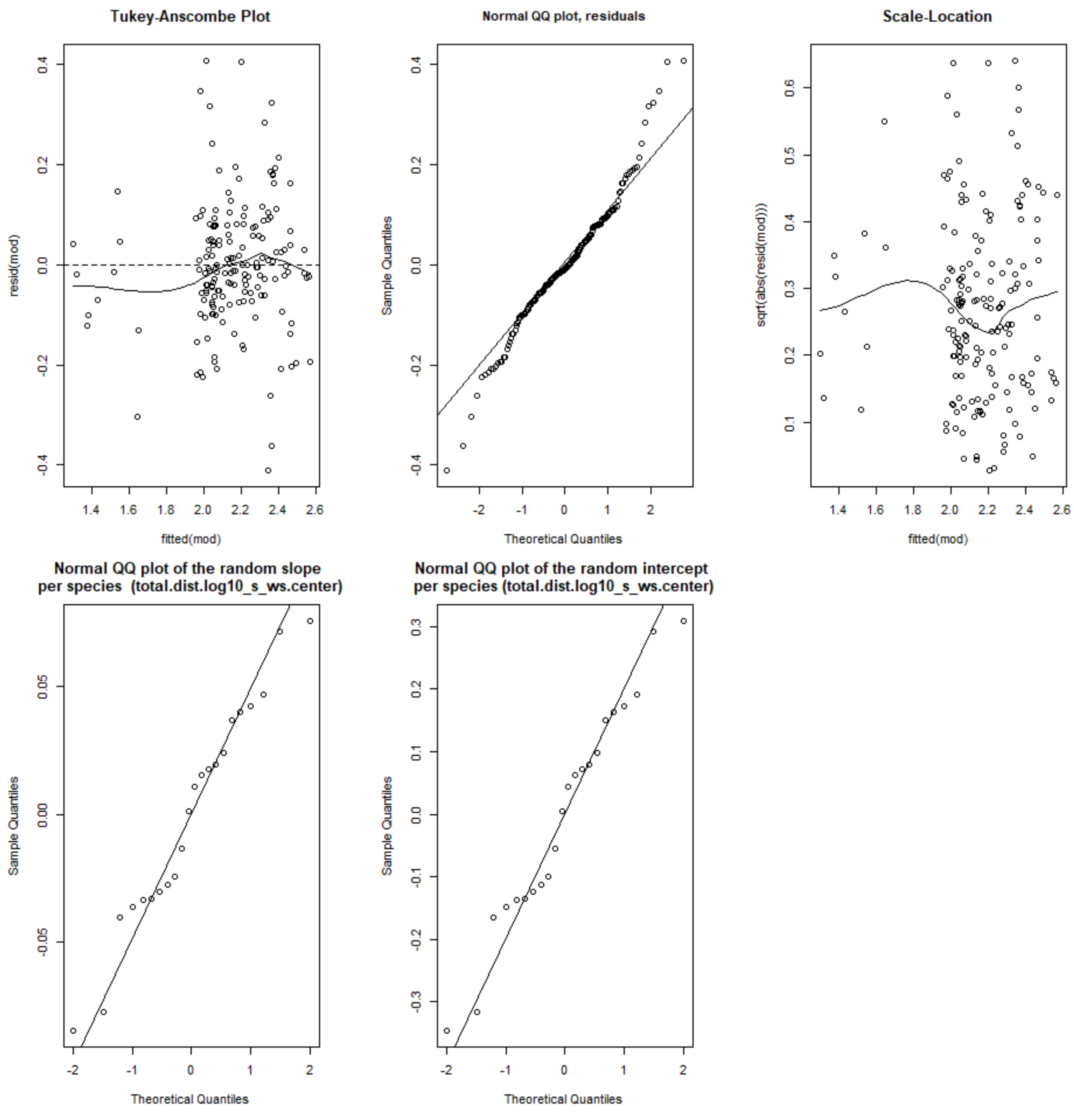


```
# --> no violation

# c) drawing conclusions
nsim <- 5000
set.seed(0470)    # specify the seed (starting value for random generator)
bsim <- sim(mod, n.sim=nsim)
colnames(bsim@fixef) <- names(fixef(mod))
r.fixef <- round(apply(bsim@fixef, 2, quantile, prob=c(0.025,0.5,0.975)),2)
r.fixef
```

```
##      (Intercept) total.dist.log10_ws.center total.dist.log10_s.mean
## 2.5%       -2.25                       0.54                    0.47
## 50%        -0.92                       0.73                    0.83
## 97.5%       0.42                       0.92                    1.19

# number of individuals considered
length(mod@resp$y)

## [1] 180

# model output
mod

## Linear mixed model fit by REML ['lmerMod']
## Formula:
## t.speed.log10 ~ total.dist.log10_ws.center + total.dist.log10_s.mean +
##     (total.dist.log10_ws.center | ID)
##    Data: s.dat
## REML criterion at convergence: -151.9979
## Random effects:
##  Groups   Name                       Std.Dev. Corr
##  ID       (Intercept)                0.19097
##           total.dist.log10_ws.center 0.04688  1.00
##  Residual                            0.13267
## Number of obs: 180, groups:  ID, 22
## Fixed Effects:
##                (Intercept)  total.dist.log10_ws.center
##                    -0.8879                      0.7298
##    total.dist.log10_s.mean
##                     0.8210

# marginal and conditional R-square
r.squaredGLMM(mod)

##            R2m       R2c
## [1,] 0.3877163 0.8007718
```

5.3. Autumn model

```
# a) modelling
mod <- lmer(t.speed.log10 ~  total.dist.log10_ws.center +
                         total.dist.log10_s.mean +
                         (total.dist.log10_ws.center|ID),
                         a.dat,
                         lmerControl(optimizer ='optimx', optCtrl=list(method='L-BFGS-B')),
                         REML = TRUE)


# b) assessing model's assumptions


# compare plot to simulated data
```

```
par(mar=rep(4,4))
compareqqnorm(mod)
```

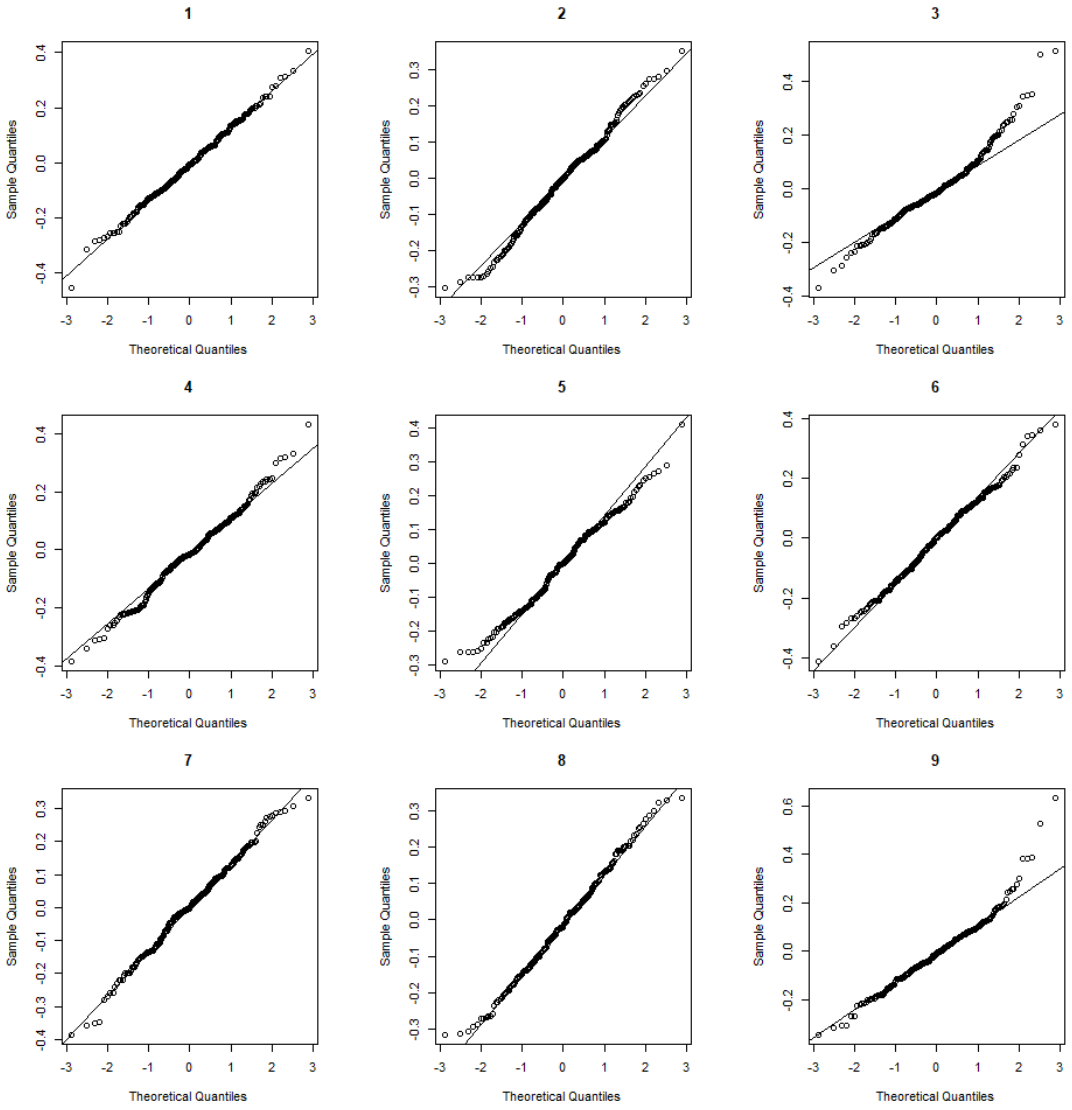


```
## [1] 3

# --> no violation

# autocorrelation
par(mfrow=c(1,2), mar=rep(4,4))
acf(resid(mod))
acf(resid(mod),type="p")
```

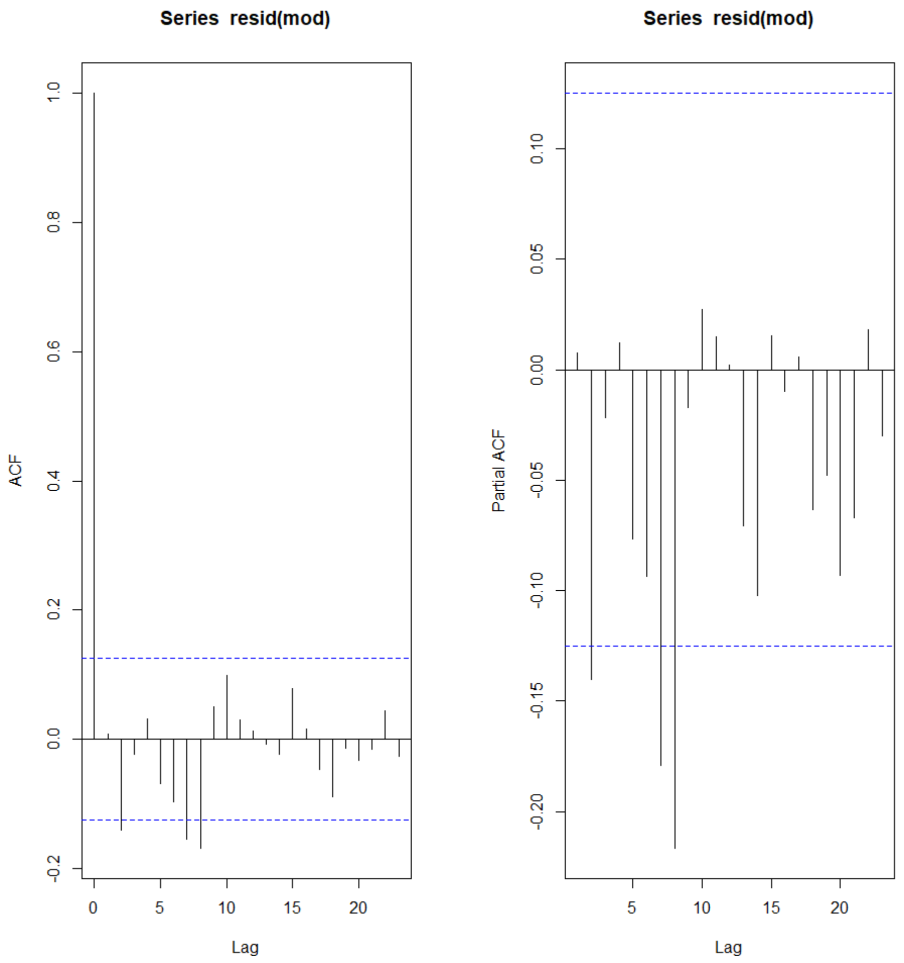


```
# --> no violation

# residual plots
t.cex <- 1
par(mfrow=c(2,3), mar=rep(4,4), mgp=c(3,1,0))
# Tukey-Ascombe plot: Residuals over predicted values
scatter.smooth(fitted(mod), resid(mod), main="Tukey-Anscombe Plot", cex=t.cex)
abline(h=0, lty=2) # residuals vs. fitted

# normal QQ plot of the residuals
qqnorm(resid(mod), main="Normal QQ plot, residuals", cex.main=t.cex) # qq of residuals
qqline(resid(mod))

# square-root of the absolute values of the residuals versus fitted values
scatter.smooth(fitted(mod), sqrt(abs(resid(mod))), main ="Scale-Location") # res. var vs. fitted

# several QQ plots of random slope and intercept
qqnorm(ranef(mod)$ID[,2], main="")
qqline(ranef(mod)$ID[,2])
title(main="Normal QQ plot of the random slope \nper species (total.dist.log10_s_ws.center)",
```

```
    cex=t.cex)

qqnorm(ranef(mod)$ID[,1], main="")
qqline(ranef(mod)$ID[,1])
title(main="Normal QQ plot of the random intercept \nper species (total.dist.log10_s_ws.center)",
    cex=t.cex)
plot.new()
```

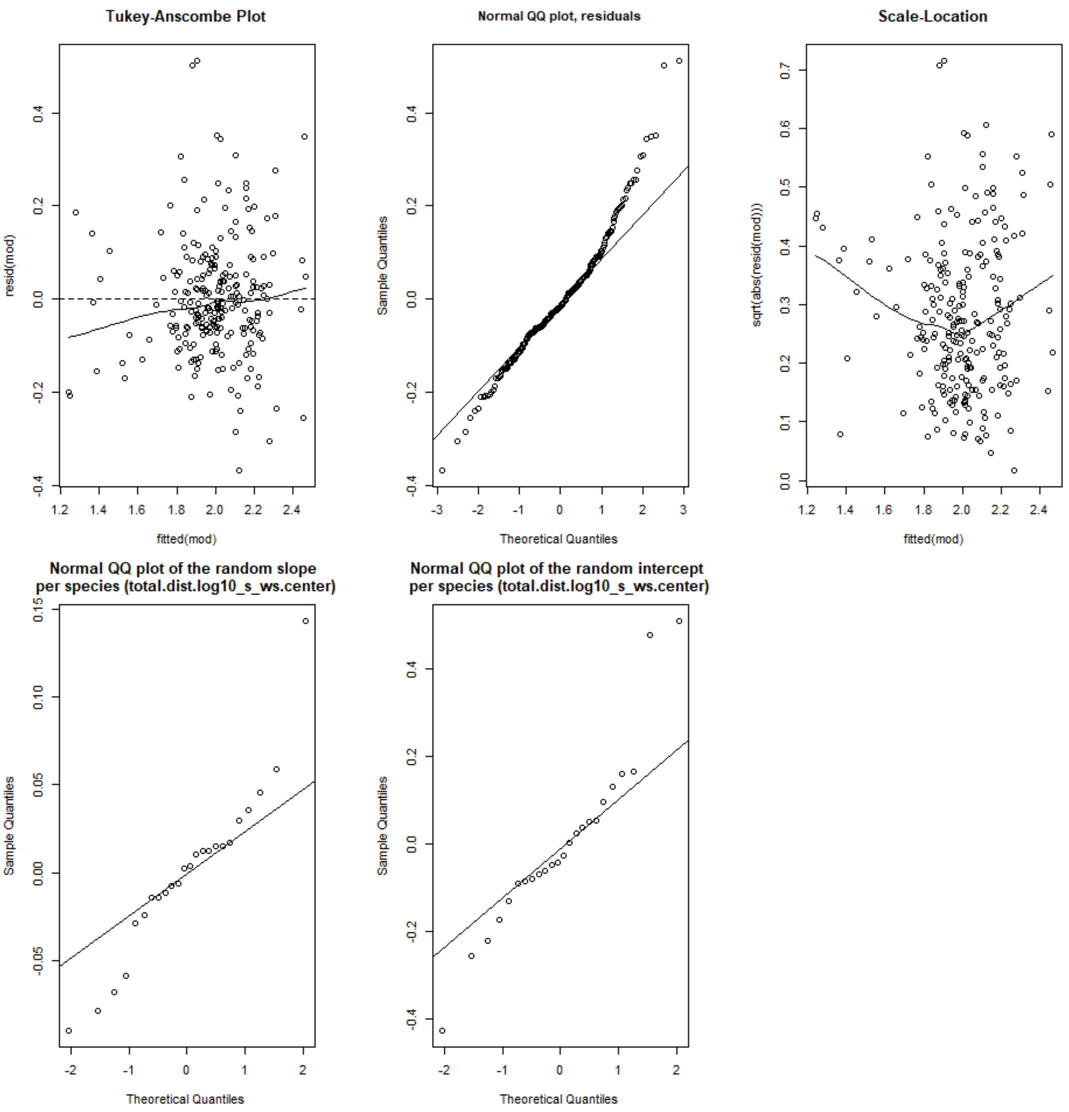


```
# --> no violation

# c) drawing conclusions
nsim <- 5000
set.seed(0470)    # specify the seed (starting value for random generator)
bsim <- sim(mod, n.sim=nsim)
colnames(bsim@fixef) <- names(fixef(mod))
r.fixef <- round(apply(bsim@fixef, 2, quantile, prob=c(0.025,0.5,0.975)),2)
r.fixef
```

```
##      (Intercept) total.dist.log10_ws.center total.dist.log10_s.mean
## 2.5%       -0.59                       0.72                   -0.02
## 50%         0.74                       0.96                    0.34
## 97.5%       2.07                       1.19                    0.70
```

```
# number of individuals considered
length(mod@resp$y)
```

```
## [1] 245
```

```
# model output
mod
```

```
## Linear mixed model fit by REML ['lmerMod']
## Formula:
## t.speed.log10 ~ total.dist.log10_ws.center + total.dist.log10_s.mean +
##     (total.dist.log10_ws.center | ID)
##    Data: a.dat
## REML criterion at convergence: -206.4836
## Random effects:
##  Groups   Name                       Std.Dev. Corr
##  ID       (Intercept)                0.2144
##           total.dist.log10_ws.center 0.1559   -0.23
##  Residual                            0.1346
## Number of obs: 245, groups:  ID, 24
## Fixed Effects:
##                (Intercept)  total.dist.log10_ws.center
##                     0.7280                      0.9575
##    total.dist.log10_s.mean
##                     0.3407
```

```
# marginal and conditional R-square
r.squaredGLMM(mod)
```

```
##            R2m       R2c
## [1,] 0.1796935 0.7689762
```

6. Modelling variation in total speed of migration with "lean" body mass

6.1 Generating variables

```
bm.dat <- ddply(dat, c("ID","breed.lat","season"), summarize,
                mean.t.speed = round(mean(t.speed, na.rm=TRUE),2),
                mean.t.speed_log10 = round(log10(mean(t.speed, na.rm=TRUE)),2),
                se.t.speed = sd(t.speed, na.rm=TRUE)/sqrt(length(which(!is.na(t.speed)))),
                bodymass = round(mean(as.numeric(bodymass), na.rm=T),2),
                bodymass_log10 = round(log10(mean(as.numeric(bodymass), na.rm=T)),2),
                t.col = min(t.col),
                family = unique(family))
```

6.2 Modelling

```
# a) modeling
mod <- lmer(mean.t.speed_log10 ~ bm_min_log10 + season +
                    (1|family),
                    bm.dat,
                    REML=FALSE,
                    weights=ifelse(is.na(bm.dat$se.t.speed), 1/mean(bm.dat$se.t.speed, na.rm=T),1/bm.
dat$se.t.speed))

# This model was initially run with the two-way interaction "bodymass_log10:season". The corresponding
95% CrI (-0.16, 0.347) included zero and was therefore removed.

# b) assessing model's assumptions
# compare plot to simulated data
par(mar=rep(4,4))
compareqqnorm(mod)
```

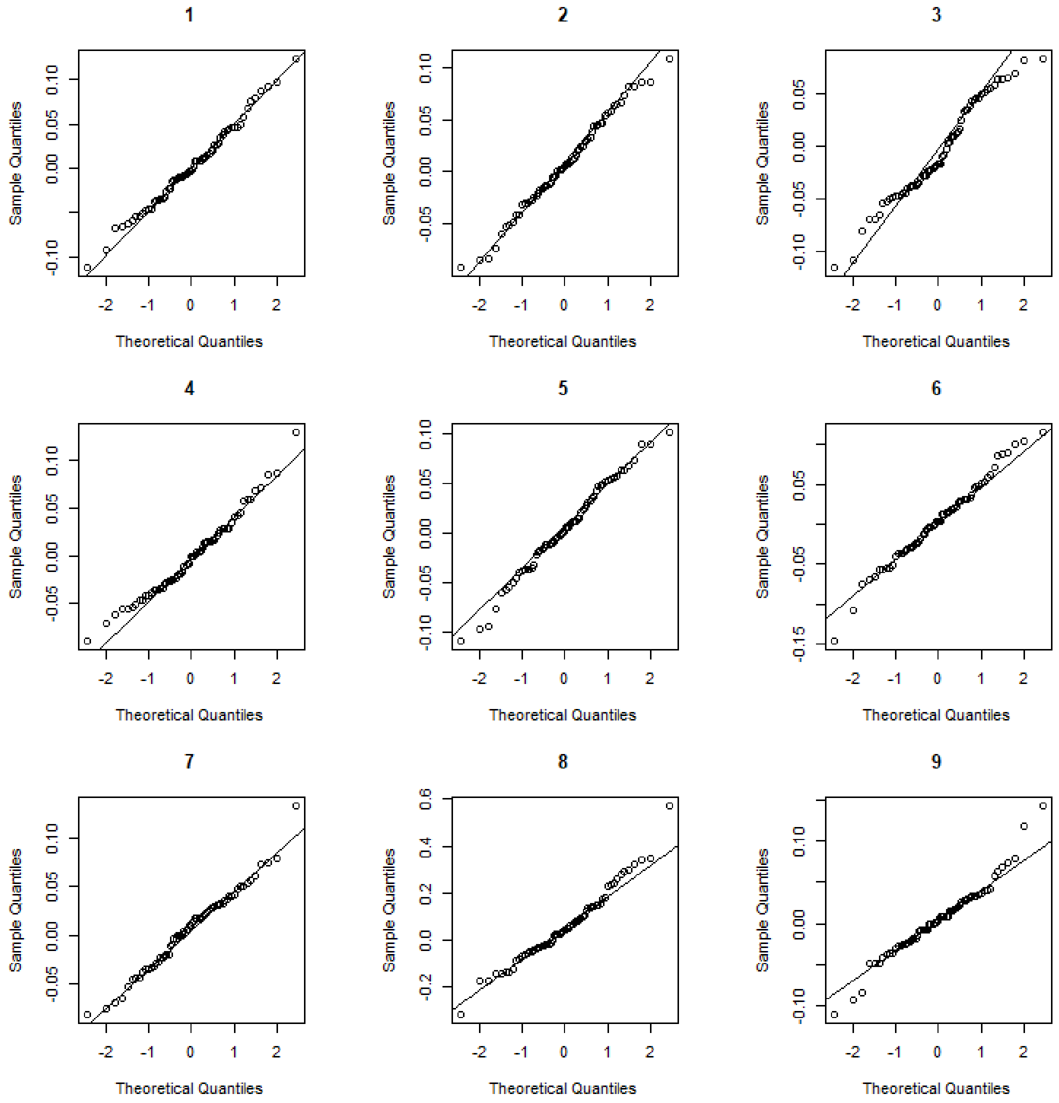

```
## [1] 8

# --> no violation

# autocorrelation
par(mfrow=c(1,2), mar=rep(4,4))
acf(resid(mod))
acf(resid(mod),type="p")
```

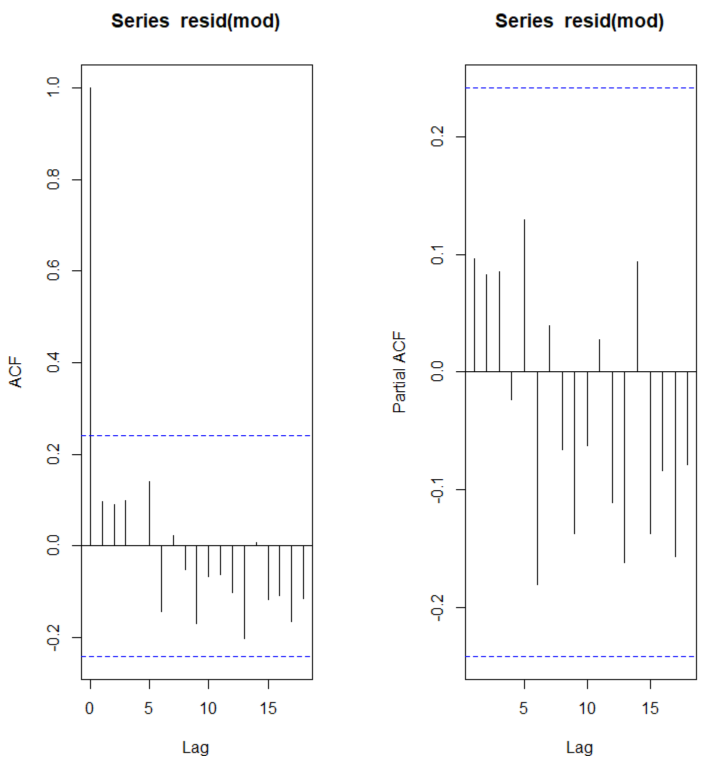


```
# --> no violation

# residual plots
t.cex <- 1
par(mfrow=c(2,2), mar=rep(4,4), mgp=c(3,1,0))
# Tukey-Ascombe plot: Residuals over predicted values
scatter.smooth(fitted(mod), resid(mod), main="Tukey-Anscombe Plot", cex=t.cex)
abline(h=0, lty=2) # residuals vs. fitted

# normal QQ plot of the residuals
qqnorm(resid(mod), main="Normal QQ plot, residuals", cex.main=t.cex) # qq of residuals
qqline(resid(mod))

# square-root of the absolute values of the residuals versus fitted values
scatter.smooth(fitted(mod), sqrt(abs(resid(mod))), main = "Scale-Location") # res. var vs. fitted

# several QQ plots of random intercept
```

```
qqnorm(ranef(mod)$family[,1], main="")
qqline(ranef(mod)$family[,1])
title(main="Normal QQ plot of the random intercept \nper family",
      cex=t.cex)
```

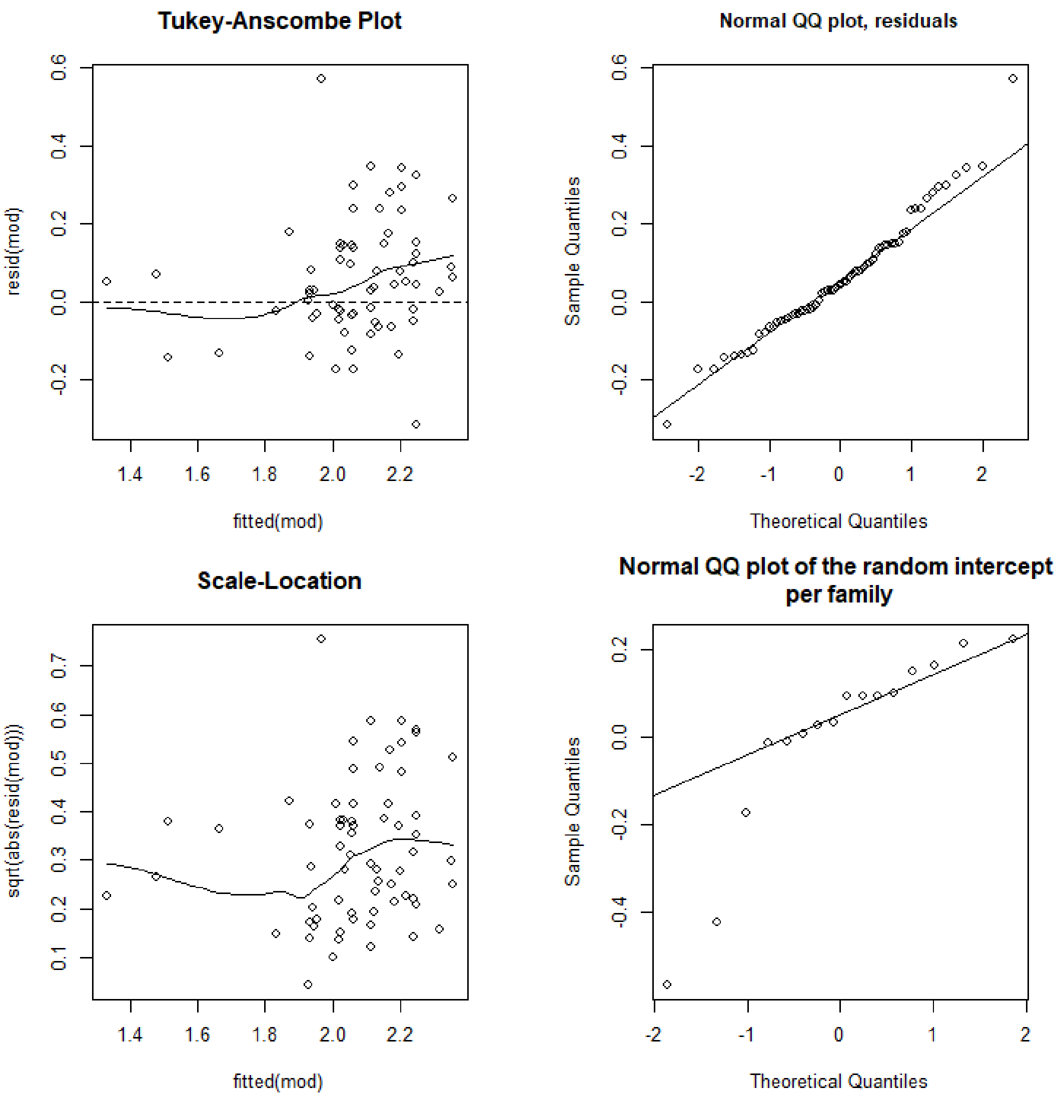


```
# --> no violation

# c) drawing conclusions
nsim <- 5000
set.seed(0470)   # specify the seed (starting value for your random generator)
bsim <- sim(mod, n.sim=nsim)
colnames(bsim@fixef) <- names(fixef(mod))
r.fixef <- round(apply(bsim@fixef, 2, quantile, prob=c(0.025,0.5,0.975)),2)
r.fixef

##      (Intercept) bm_min_log10 seasonspr
## 2.5%        1.34        -0.15      0.11
```

```
## 50%        1.75       0.13     0.18
## 97.5%       2.15       0.41     0.26

# number of individuals considered
length(mod@resp$y)

## [1] 66

# model output
mod

## Linear mixed model fit by maximum likelihood  ['lmerMod']
## Formula: mean.t.speed_log10 ~ bm_min_log10 + season + (1 | family)
##    Data: bm.dat
## Weights:
## ifelse(is.na(bm.dat$se.t.speed), 1/mean(bm.dat$se.t.speed, na.rm = T),
##     1/bm.dat$se.t.speed)
##      AIC      BIC   logLik deviance df.resid
##  -9.8537   1.0946   9.9268 -19.8537       61
## Random effects:
##  Groups   Name        Std.Dev.
##  family   (Intercept) 0.22457
##  Residual             0.04426
## Number of obs: 66, groups:  family, 16
## Fixed Effects:
##  (Intercept)  bm_min_log10     seasonspr
##       1.7513        0.1239        0.1845

# marginal and conditional R-square
r.squaredGLMM(mod)

##           R2m       R2c
## [1,] 0.165578 0.9687947
```

7. Controlling for shared ancestry

Comparative analyses including different species require to control for the effect of species' phylogenetic relationships (Felsenstein, J. 1985 Phylogenies and the comparative method. Am. Nat. 125, 1-15). The phylogenetic tree of the species involved was derived from TIMETREE (http://timetree.org; Kumar, S., Stecher, G., Suleski, M. & Hedges, S. B. 2017 TimeTree: A resource for timelines, timetrees, and divergence times. Mol. Ecol. Evol. 34, 1812-1819 and Hedges, S. B., Martin, J., Suleski, M., Paymer, M. & Kumar, M. S. 2015 Tree of life reveals clock-like speciation and diversification. Mol. Ecol. Evol. 32, 835-845). The variation in total speed of migration was modelled using a generalized least squares regression model, function "gls" of the R packages "nlme" (Pinheiro, J., Bates, D., DebRoy, S., Sarkar, D. & Team, R. C. 2017 nlme: Linear and Nonlinear Mixed Effects Models. R package, https:// CRAN.R-project.org/package=nlme). This model allows correlated errors and unequal variances. This model was run with speciec-specific data, but not with population-specific data, because the phylogenetic relationship only provides a model for expected covariation on the species level. Having different populations and not

species as tips in the phylogeny would involve arbitrary assumptions about the variation between populations.

7.1 Spring

```
# reading tree data
tree.dat <- read.tree("species list songbirds body mass.nwk.txt")

# dropping species for which no spring data are available
tree.dat.1 <- drop.tip(tree.dat, "Saxicola_torquata")
tree.dat.2 <- drop.tip(tree.dat.1, "Phylloscopus_trochilus")

# adjusting data set
dat$ID.a <- as.character(dat$ID)
# give the two northern wheatear populations the same name
dat$ID.a[dat$ID.a=="northern wheatear AK"] <- "northern wheatear"
dat$ID.a[dat$ID.a=="northern wheatear SE"] <- "northern wheatear"
dat$ID.a <- as.factor(dat$ID.a)


# generating data for spring
s.bm <- ddply(dat[dat$season=="spr",], c("ID.a"), summarize,
        n = length(na.omit(t.speed)),
        mean.t.speed = round(mean(t.speed, na.rm=TRUE),2),
        mean.t.speed_log10 = round(log10(mean(t.speed, na.rm=TRUE)),2),
        se.t.speed = sd(t.speed, na.rm=TRUE)/sqrt(length(which(!is.na(t.speed)))),
        var.t.speed = var(t.speed, na.rm=TRUE)/sqrt(length(which(!is.na(t.speed)))),
        bodymass = round(mean(as.numeric(bodymass), na.rm=T),2),
        bodymass_log10 = round(log10(mean(as.numeric(bodymass), na.rm=T)),2),
        t.col = min(t.col),
        family = unique(family))

# including scientific names
s.bm$snames <- c("Hirundo_rustica","Setophaga_striata","Sturnus_philippensis","Calcarius_ornatus","C
uculus_canorus","Tyrannus_tyrannus","Coracias_garrulus","Vermivora_chrysoptera","Acrocephalus_aru
ndinaceus","Carduelis_cannabina","Oenanthe_oenanthe","Emberiza_hortulana","Ficedula_hypoleuca","
Lanius_collurio","Vireo_olivaceus","Luscinia_svecica","Tyrannus_forficatus","Ficedula_semitorquata","Pl
ectrophenax_nivalis","Catharus_ustulatus","Anthus_campestris","Catharus_fuscescens","Tyrannus_verti
calis","Hylocichla_mustelina")

# give row names
row.names(s.bm) <- s.bm$snames

# order data as in tree data
t.order <- match(tree.dat.2$tip.label,row.names(s.bm))
s.bm <-s.bm[t.order,]

# modelling by expected covariance under a Brownian model
modB1 <- gls(mean.t.speed_log10 ~ bodymass_log10,
       data = s.bm,
```

```
        correlation = corBrownian(value = 1, phy = tree.dat.2),
        control=glsControl(opt = "optim"),
        method = "ML")

# residual anaylsis
mod <- modB1
par(mar=rep(5,4))
plot(mod, resid(., type="n")~fitted(.), main="Normalized Residuals v Fitted Values",
   abline=c(0,0),  cex=1.5, pch=19, cex.axis=1.5, cex.lab=1.5)
```

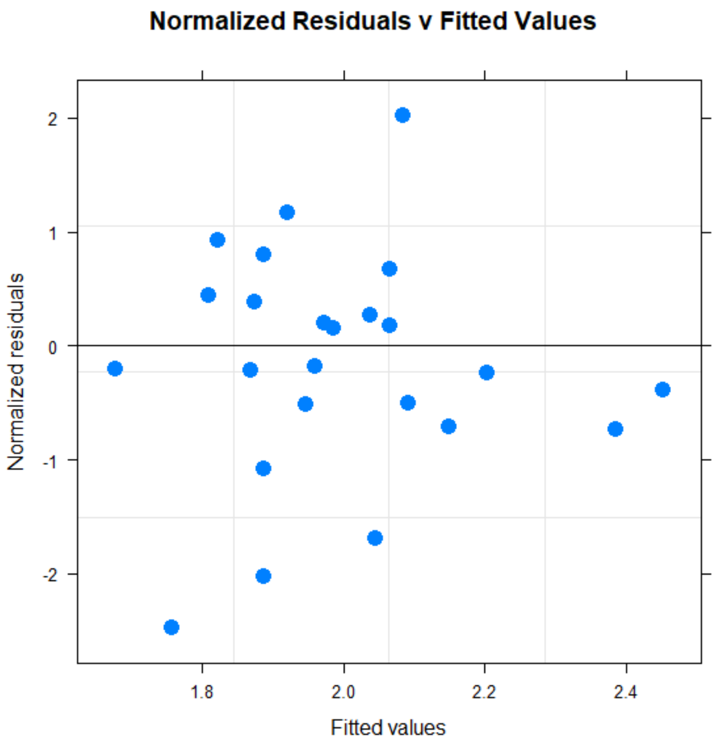


```
res <- resid(mod, type="n"); qqnorm(res, cex=1.5, pch=19, cex.axis=1.5, cex.lab=1.5); qqline(res)
```

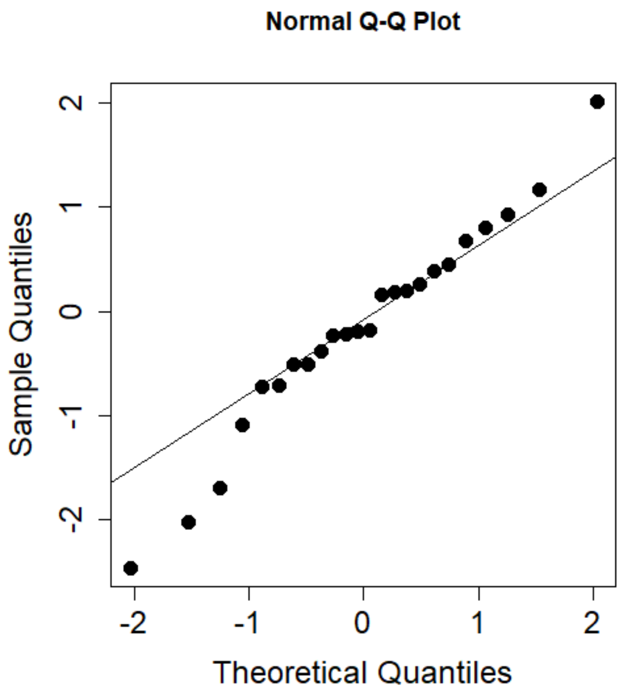


```
# --> no violation

# drawing conclusions
summary(mod)

## Generalized least squares fit by maximum likelihood
##   Model: mean.t.speed_log10 ~ bodymass_log10
##   Data: s.bm
##        AIC      BIC    logLik
##   23.11949 26.65365 -8.559743
##
## Correlation Structure: corBrownian
##  Formula: ~1
##  Parameter estimate(s):
## numeric(0)
##
## Coefficients:
##                    Value Std.Error  t-value p-value
## (Intercept)    1.0349428 0.7361084 1.405965  0.1737
## bodymass_log10 0.6560185 0.3839841 1.708452  0.1016
##
##  Correlation:
##                (Intr)
## bodymass_log10 -0.916
##
## Standardized residuals:
##        Min         Q1        Med         Q3        Max
```

```
## -0.90661154  0.06753008  0.52290853  0.68807030  1.18689148
##
## Residual standard error: 0.5711011
## Degrees of freedom: 24 total; 22 residual

# The linear model fit using generalized least square and controlling for shared ancestry does not provide a different effect of body mass on total speed of migration than detailed in 7.2.
```

7.2 Autumn

```
# reading tree data
tree.dat <- read.tree("species list songbirds body mass.nwk.txt")

# dropping species for which no autumn data are available
tree.dat.1 <- drop.tip(tree.dat, "Hylocichla_mustelina")

# adjusting data set
dat$ID.a <- as.character(dat$ID)
# giving the two northern wheatear populations the same name
dat$ID.a[dat$ID.a=="northern wheatear AK"] <- "northern wheatear"
dat$ID.a[dat$ID.a=="northern wheatear SE"] <- "northern wheatear"
dat$ID.a <- as.factor(dat$ID.a)
# excluding wood thrush, because there is no estimate for total speed of migration
dat <- dat[dat$ID.a!="wood thrush",]
dat$ID.a <- as.factor(dat$ID.a)

# generating data for autumn
a.bm <- ddply(dat[dat$season == "aut",], c("ID.a"), summarize,
        n = length(na.omit(t.speed)),
        mean.t.speed = round(mean(t.speed, na.rm=TRUE),2),
        mean.t.speed_log10 = round(log10(mean(t.speed, na.rm=TRUE)),2),
        se.t.speed = sd(t.speed, na.rm=TRUE)/sqrt(length(which(!is.na(t.speed)))),
        var.t.speed = var(t.speed, na.rm=TRUE)/sqrt(length(which(!is.na(t.speed)))),
        bodymass = round(mean(as.numeric(bodymass), na.rm=T),2),
        bodymass_log10 = round(log10(mean(as.numeric(bodymass), na.rm=T)),2),
        t.col = min(t.col),
        family = unique(family))



# including scientific names
a.bm$snames <- c("Hirundo_rustica","Setophaga_striata","Sturnus_philippensis","Calcarius_ornatus","Cuculus_canorus","Tyrannus_tyrannus","Coracias_garrulus","Vermivora_chrysoptera","Acrocephalus_arundinaceus","Carduelis_cannabina","Oenanthe_oenanthe","Emberiza_hortulana","Ficedula_hypoleuca","Lanius_collurio","Vireo_olivaceus","Luscinia_svecica","Tyrannus_forficatus","Ficedula_semitorquata","Plectrophenax_nivalis","Saxicola_torquata","Catharus_ustulatus","Anthus_campestris","Catharus_fuscescens","Tyrannus_verticalis","Phylloscopus_trochilus")

# give row names
row.names(a.bm) <- a.bm$snames
```

```
# order data as in tree data
t.order <- match(tree.dat.1$tip.label,row.names(a.bm))
a.bm <-a.bm[t.order,]

# modelling by expected covariance under a Brownian model
modB1 <- gls(mean.t.speed_log10 ~ bodymass_log10,
        data = a.bm,
        correlation = corBrownian(value = 1, phy = tree.dat.1),
        control=glsControl(opt = "optim"),
        method = "ML")

# residual anaylsis
mod <- modB1
par(mar=rep(5,4))
plot(mod, resid(., type="n")~fitted(.), main="Normalized Residuals v Fitted Values",
   abline=c(0,0),  cex=1.5, pch=19, cex.axis=1.5, cex.lab=1.5)
```

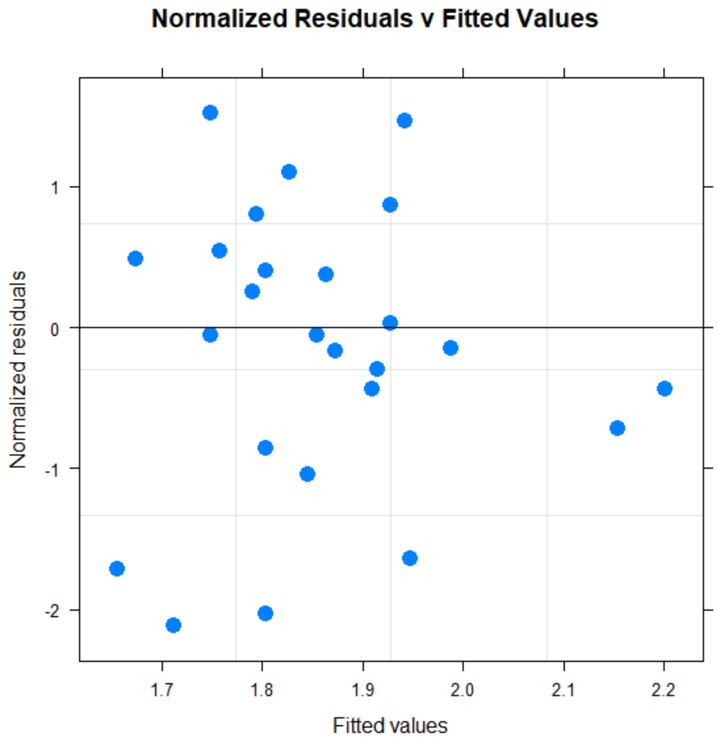


```
res <- resid(mod, type="n"); qqnorm(res, cex=1.5, pch=19, cex.axis=1.5, cex.lab=1.5); qqline(res)
```

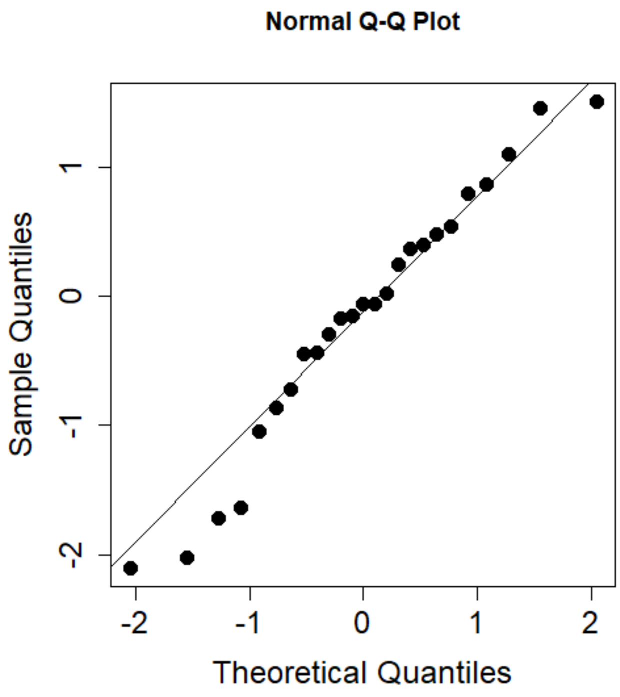


```
# --> no violation

# drawing conclusions
summary(mod)

## Generalized least squares fit by maximum likelihood
##   Model: mean.t.speed_log10 ~ bodymass_log10
##   Data: a.bm
##        AIC      BIC    logLik
##   16.49155 20.14818 -5.245776
##
## Correlation Structure: corBrownian
##  Formula: ~1
##  Parameter estimate(s):
## numeric(0)
##
## Coefficients:
##                    Value Std.Error  t-value p-value
## (Intercept)    1.2039821 0.6115501 1.968738  0.0611
## bodymass_log10 0.4615032 0.3257711 1.416649  0.1700
##
##  Correlation:
##                (Intr)
## bodymass_log10 -0.915
##
## Standardized residuals:
##        Min         Q1        Med         Q3        Max
```

```
## -0.88219335  0.08306794  0.40160137  0.67852519  1.64696224
##
## Residual standard error: 0.4805479
## Degrees of freedom: 25 total; 23 residual
```

# The linear model fit using generalized least square and controlling for shared ancestry does not provide a different effect of body mass on total speed of migration than detailed in 6.2.
